# Intelligent design of mechanical metamaterials: a GCNN-based structural genome database approach

**DOI:** 10.1093/nsr/nwaf053

**Published:** 2025-02-20

**Authors:** Wenyu Hao, Zongliang Du, Xiuquan Hou, Yilin Guo, Chang Liu, Weisheng Zhang, Huajian Gao, Xu Guo

**Affiliations:** State Key Laboratory of Structural Analysis, Optimization and CAE Software for Industrial Equipment, Department of Engineering Mechanics, Dalian University of Technology, Dalian 116023, China; State Key Laboratory of Structural Analysis, Optimization and CAE Software for Industrial Equipment, Department of Engineering Mechanics, Dalian University of Technology, Dalian 116023, China; Ningbo Institute of Dalian University of Technology, Ningbo 315016, China; Institute of Artificial Intelligence and Robotics, Xi’an Jiaotong University, Xi’an 710049, China; State Key Laboratory of Structural Analysis, Optimization and CAE Software for Industrial Equipment, Department of Engineering Mechanics, Dalian University of Technology, Dalian 116023, China; State Key Laboratory of Structural Analysis, Optimization and CAE Software for Industrial Equipment, Department of Engineering Mechanics, Dalian University of Technology, Dalian 116023, China; Ningbo Institute of Dalian University of Technology, Ningbo 315016, China; State Key Laboratory of Structural Analysis, Optimization and CAE Software for Industrial Equipment, Department of Engineering Mechanics, Dalian University of Technology, Dalian 116023, China; Ningbo Institute of Dalian University of Technology, Ningbo 315016, China; Mechano-X Institute, Applied Mechanics Laboratory, Department of Engineering Mechanics, Tsinghua University, Beijing 100084, China; State Key Laboratory of Structural Analysis, Optimization and CAE Software for Industrial Equipment, Department of Engineering Mechanics, Dalian University of Technology, Dalian 116023, China; Ningbo Institute of Dalian University of Technology, Ningbo 315016, China

**Keywords:** mechanical metamaterial, structural genome database, moving morphable component, graph convolutional neural network, structure-property mapping

## Abstract

The reciprocal mapping between the geometry and properties of a unit cell is crucial for the intelligent and inverse design of advanced materials and structural systems. Beyond classical homogenization-based numerical methods, this paper presents an efficient and accurate mapping between the geometry and properties of a class of unit cells described by moving morphable components, achieved via a graph convolutional neural network. This leads to a structural genome database (SGD) approach for the intelligent design of mechanical metamaterials. Using the SGD approach, metamaterials exhibiting the Hashin–Shtrikman upper bound of bulk modulus, auxetic behavior and the unimodal property have been created, with design efficiency improved by 3–4 orders of magnitude. Additionally, transfer learning and a small amount of training data allow the SGD to predict non-local behaviors beyond a unit cell, such as optimized unit cells with critical buckling strength enhanced by nearly 200% and a bandgap metamaterial with a relative bandgap width of 51%. Experimentally validated optimized metamaterials demonstrate auxetic behavior and superior buckling resistance. The proposed SGD approach holds promise for the advanced design of multi-scale and multi-physics systems.

## Teaser

Combining the explicit topology optimization and graph convolutional neural network, a structural genome database is constructed and provides a unified design toolbox for various mechanical metamaterials.

## INTRODUCTION

Metamaterials are architected materials that exhibit extraordinary macroscopic properties such as negative Poisson’s ratio [1], zero/negative thermal expansions [2], extreme elastic moduli [3], topologically protected properties [4] and programmable functional properties [5], achieved through rational design of unit cells. For mechanical metamaterials, the bulk modulus and Poisson’s ratio are key effective properties that can be systematically designed using topology optimization and homogenization theories [6–9]. These have applications in sports protection [10], flexible electronics design [11], bone scaffolds and implants [12], among others. The rapid advancements in additive manufacturing have enabled the fabrication of metamaterials with intricate unit cells and tailored properties [13].

In traditional structural topology optimization methods, the solution process typically involves tens to hundreds of iterative analyses and updates of intermediate designs. Improving the efficiency of topology optimization using modern techniques such as machine learning is an intriguing area of research [14–23]. Additionally, since unit cells with a homogenized effective property are not unique, solving non-convex inverse homogenization problems presents a significant challenge.

To accelerate the design process, the deep learning-enhanced design of mechanical metamaterials has emerged as a prominent research topic [24]. Related work can be mainly categorized into three classes.

1. *Property prediction.* This involves establishing an implicit mapping between structural parameters and effective properties using a discriminative model, which speeds up the screening process of metamaterials [25,26].
2. *Accelerated optimization.* Well-trained discriminative models serve as surrogates in inverse design, replacing the more expensive finite-element analysis. This significantly reduces the time required for the design process [27–33].
3. *Generative design.* Generative models directly generate structures based on certain conditions, enhancing the diversity of designs [34–36].

Although promising progress has been achieved in those works, there are however limitations related to structural description methods and sample generation techniques, resulting in a relatively limited design space in both configuration and property spaces of the unit cells. Consequently, most of the related studies focused on a specific type of mechanical metamaterial, and there lacks a unified deep learning model applicable to various mechanical metamaterials.

In biology, the genome encompasses all the genetic information of an organism. Once the genome of an organism is determined, its characteristics can be selectively controlled through gene screening. Similarly, in metamaterials, the unit cell can be seen as the ‘gene’, and the corresponding effective property as the ‘character’. Inspired by the success of the human genome database [37,38], a structural genome database (SGD), which collects configurations of unit cells and their effective properties, can be established and used for the intelligent design of advanced materials and structures. To construct a representative SGD, we need to address (a) how to effectively describe the structural genomes in both two and three dimensions, (b) how to achieve efficient and accurate reciprocal mapping between the structural genes and their properties and (c) how the representativeness of the SGD can be evaluated.

In the present work, the moving morphable component (MMC) method [39], which utilizes a set of components with their locations and shapes described by geometry parameters as the building blocks, is employed to describe two-dimensional (2D) and 3D unit cells (i.e. structural genes). This method leverages the advantages of fewer design variables, an explicit geometry description and decoupling between the topology description and finite-element discretization. Additionally, the geometrical connections of structural components are naturally suitable for graph neural network (GNN) [40], where input data are generated from non-Euclidean space and represented as graphs with complex relationships and interdependencies between objects [41]. By adopting the upgraded graph convolutional neural network (GCNN) [42], which introduces the convolution operator into the GNN to aggregate input features more effectively, an accurate machine learning model can be developed to predict the underlying relationship between MMC parameters (more details can be found in Sections B and C within the online supplementary material) and the effective properties of structural genes.

Specifically, with the structural genes described by the MMC method and their effective elastic tensors predicted by the GCNN model, an SGD is constructed by randomly generating 240 000 samples for 2D unit cells and 100 000 samples for 3D unit cells. To validate the completeness of the structural genome as a representative database, the achievable bounds of the bulk modulus of the structural genes are compared with their theoretical values. Furthermore, the GCNN model and optimizer are combined to bridge the targeted effective elastic tensor and the geometry parameters of structural genes. In this way, the proposed SGD provides an efficient and accurate reciprocal mapping between geometry parameters and effective elastic tensors, serving as a representative database for the rapid screening of mechanical metamaterials with target properties, such as unit cells with maximized bulk modulus/shear modulus, auxetic materials and unimode materials [43].

The proposed SGD, combining the MMC-based topology description method and GCNN model, offers several advantages.

1. The samples in the database are described by an explicit topology description, providing a relatively complete coverage of effective elastic properties.
2. It can be used as a unified deep learning model for efficient design of various mechanical metamaterials, e.g. metamaterials with extreme modulus, and even metamaterials with non-local behavior such as superior buckling resistance and a broad bandgap at low frequency.
3. While maintaining high accuracy, the design efficiency of mechanical metamaterials is improved by 3–4 orders of magnitude using the SGD.
4. Thanks to the explicit geometry description, fabrication uncertainties can be easily considered, and the optimized metamaterials can be directly modeled in CAD/CAE software for further applications.

The concept of a data-driven structural genome database may pave the way for AI-enhanced design of metamaterials and advanced materials/structures.

## RESULTS

### Summary of the SGD

**Figure 1.**
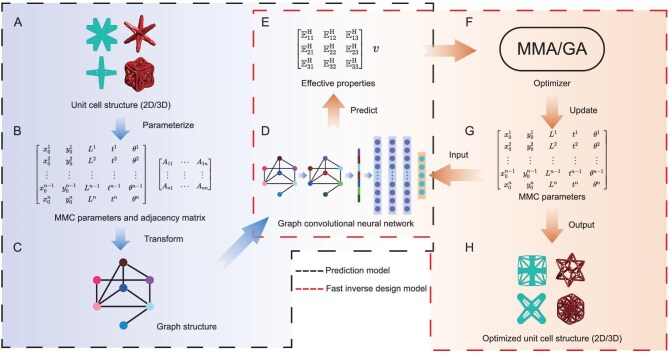
A schematic illustration of the architecture of the proposed SGD.

Establishing a reciprocal mapping between geometry parameters and effective properties of unit cells, as illustrated in Fig. 1, the proposed SGD comprises two models.

1. *Prediction model.* Based on the GCNN model (enclosed by black dotted lines, i.e. modules A-B-C-D-E). Specifically, the MMC-described unit cells shown in module A are first converted into the feature matrix composed of MMC parameters and the adjacency matrix in module B. In module C, the feature matrix and adjacency matrix form a graph structure that serves as the input for the GCNN, illustrated in module D. Without employing the finite-element method (FEM) or other numerical analysis procedures, the well-trained GCNN predicts the effective properties (e.g. elasticity tensor and volume fraction) with high accuracy and efficiency.
2. *Fast inverse design model.* This model combines the GCNN and the optimizer (e.g. moving asymptotic algorithm (MMA) [44] or genetic algorithm (GA) [45]) to obtain unit cells with target effective properties (enclosed by red dotted lines, i.e. modules G-D-E-F-G-H). Starting with the default or given initial design in module G, the graph structure is first produced. Then the objective function and volume constraint in module E are predicted using the GCNN model in module D. If the gradient-based optimizer is adopted, the sensitivity information is also obtained via the back-propagation (BP) algorithm of the GCNN model. The optimizer in module F then updates the design variables in module G. Similar to the prediction model, the effective property of the updated design is predicted using the GCNN in module D. This iterative process continues until the convergence condition is met. Utilizing the explicit description of the MMC method, the CAD model of optimized designs is directly provided in module H.

It is important to note that the cornerstone of the proposed SGD is the highly accurate and efficient prediction model.

### The prediction model based on the MMC and GCNN

**Figure 2.**
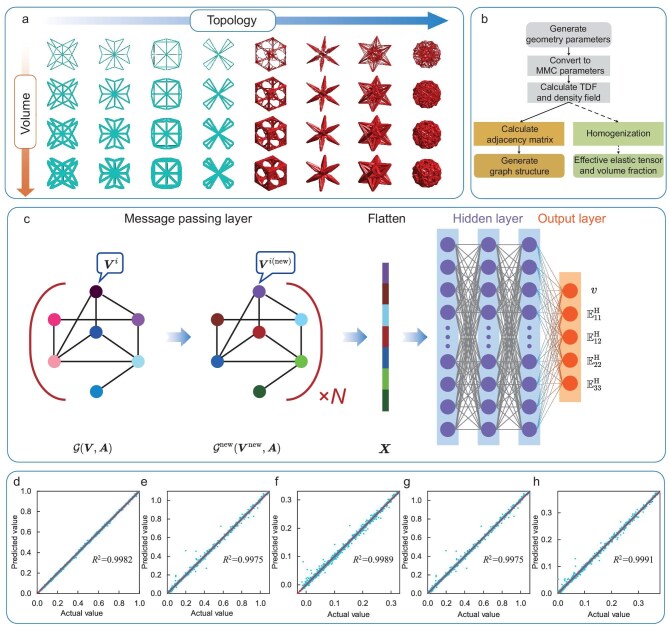
(a) Some representative unit cells in the 2D and 3D SGDs. (b) The sample generation process. (c) The architecture of the GCNN model and actual values versus the predicted values: (d) volume fraction, (e) $\mathbb {E}_{11}^{\rm H}$, (f) $\mathbb {E}_{12}^{\rm H}$, (g) $\mathbb {E}_{22}^{\rm H}$ and (h) $\mathbb {E}_{33}^{\rm H}$ in the effective elastic tensor.

As an initial attempt to construct the SGD, we focus on unit cells with specific symmetries, i.e. $C_{4v}$-symmetric 2D unit cells and cubic-symmetric 3D unit cells (as illustrated in Fig. 2a). Detailed information on the generation rules and design variables of the unit cells is provided in Sections B and C within the online supplementary material.

As illustrated in Fig. 2b, for each randomly generated sample, the geometry parameters (e.g. the locations of control points and the widths of components shown in Sections B and C within the online supplementary material) are first converted to MMC parameters. Detailed in the Methods section below, the design domain is discretized by fixed meshes, and the topology description function is calculated from the MMC parameters. The adjacency matrix is then obtained based on the connection of MMCs [46]. The effective elastic tensor is determined by the asymptotic homogenization analysis [47,48], with more details available in Section A within the online supplementary material. In the architecture of the GCNN in Fig. 2c, the feature matrix $\boldsymbol {V}$ composed of the MMC parameters (i.e. $\boldsymbol {V}^i = (x_0^i,y_0^i,L^i,t^i,{\rm {sin}}\theta ^{i},{\rm {cos}}\theta ^{i})$ corresponds to the MMC parameters of the *i*th component) and the adjacency matrix $\boldsymbol {A}$ described in the Methods section are converted to a graph structure $\mathcal {G}(\boldsymbol {V},\boldsymbol {A})$ as the input. The graph structure then passes through three message passing layers (MPLs) for feature transmission and aggregation (*N* denotes the number of MPLs, with $N=3$ in the 2D and 3D SGD cases and $N=6$ in the buckling module and bandgap module of the 2D SGD). The new graph structure $\mathcal {G}^{\rm {new}}$ is expanded to a column vector $\boldsymbol X$, which has more vital features than the original graph structure. Finally, fully connected layers are used to predict the volume fraction and effective elastic tensor of the unit cells (i.e. $\boldsymbol y= (v, \mathbb {E}_{11}^{\rm H}, \mathbb {E}_{12}^{\rm H}, \mathbb {E}_{22}^{\rm H}, \mathbb {E}_{33}^{\rm H})^\top$). The details of the GCNN are presented in Subsection E.1 within the online supplementary material.

The GCNN model is implemented using the PyTorch toolbox [49]. A total of 240 000 samples in the 2D case are randomly divided into 80%, 10% and 10% as the training set, the verification set and the test set, respectively. As the loss function, the mean-square-error (MSE) value of the 2D GCNN model converges to 0.026, and comparisons between the predicted and true values of the test set are shown in panels (d–h) of Fig. 2, where all the data points are predominantly distributed close to the line $y=x$, confirming the high accuracy of the prediction model. The 3D SGD is presented in Sections C– E within the online supplementary material. Notably, using the GCNN as a surrogate model, the classical asymptotic homogenization process can be accelerated by 3–4 orders of magnitude. In particular, Section F within the online supplementary material presents the computational cost of the finite-element analysis and SGD prediction. In the 2D case, the acceleration factor is 1050, while this value increases to $38\, 700$ for the 3D case to predict the effective elasticity tensor. The acceleration factor for predicting the critical buckling factor and the dispersion of 2D unit cells is 8516 and 3827. This is essential for a fast inverse design model that often requires ten to hundreds of iterations even using gradient-based optimization algorithms.

### The fast inverse design model with the MMA optimizer

With the help of the GCNN model, the inverse design of unit cells with target effective property corresponds to the mathematical programming


(1) \begin{eqnarray*}
\begin{array}{c}\text{find} \quad \boldsymbol {d} \in \mathcal {D}_{\rm ad} \\
\text{min.} \quad f = f(\boldsymbol {Y}) \\
\text{s. t.} \quad \boldsymbol {Y} = \mathbb {G}(\mathcal {G}\lbrace {\boldsymbol V}({\boldsymbol d}),\boldsymbol {A}({\boldsymbol d})\rbrace ), \\
V(\boldsymbol {d}) \le \bar{v}, \end{array}
\end{eqnarray*}

where $\boldsymbol {d}$ is the vector of design variables (geometry parameters of the unit cell) with its admissible set denoted by $\mathcal {D}_{\rm ad}$. Here *f* and $\boldsymbol {Y}$ denote the objective function and the output vector of the GCNN model, respectively, with its mapping denoted by $\mathbb {G}$. The volume fraction constraint is expressed by the volume fraction of the solid material *V* and the max allowable volume fraction $\bar{v}$, respectively.

During training of the GCNN, the related parameters are optimized using a gradient descent algorithm. Consequently, the sensitivity of the output vector with respect to the graph structure can be directly obtained through the BP algorithm. By applying the chain rule, the sensitivity of the objective function with respect to design variables can be written as


(2) \begin{eqnarray*}
\frac{\partial f}{\partial \boldsymbol {d}} = \frac{\partial f}{\partial \boldsymbol {Y}} \cdot \frac{\partial \boldsymbol {Y}}{\partial \boldsymbol {d}}.
\end{eqnarray*}

The MMA optimizer [44] is selected to iteratively solve the above formulation. Notably, since exact analysis (e.g. finite-element analysis) has been replaced by the GCNN model’s mapping, optimization problem (1) can also be efficiently solved using gradient-free algorithms such as the GA [45].

### Verifying the representativeness of the 2D SGD

**Figure 3.**
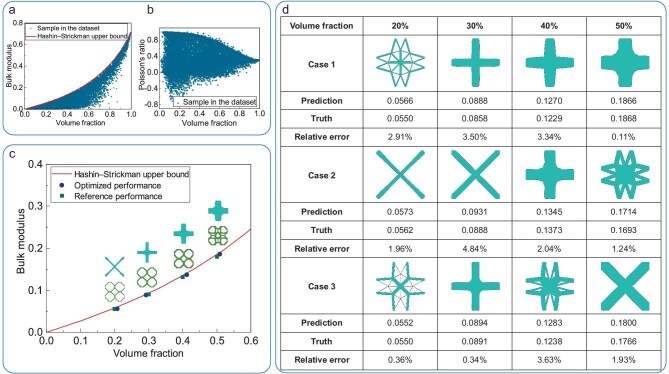
Distributions of samples in the 2D SGD: (a) the bulk modulus and (b) Poisson’s ratios. (The data points in the figure represent the bulk modulus and Poisson’s ratio of samples in the dataset at different volume fractions, with the red lines indicating the HS upper bounds. Young’s modulus $E=1$ and Poisson’s ratio $\nu =0.3$ for the solid material.) (c) Validation of the optimized unit cells with maximized bulk modulus in the 2D SGD (optimized performance depicts the exact bulk modulus of SGD designs obtained by homogenization analysis and the exact volume fraction). (d) The maximum bulk modulus designs generated by the 2D SGD according to different volume fraction constraints.

In homogenization theory, an achievable effective elasticity tensor generally corresponds to an infinite number of unit-cell types. The bulk modulus and shear modulus of the solid material are respectively given by $\kappa = {E}/{(2( {1 - \nu } ))}$ and $\mu = {E}/ {(2( {1 + \nu } ))}$, where *E* is Young’s modulus and $\nu$ is Poisson’s ratio. As long as most of the realizable effective properties are covered, such a database can be considered representative. The distributions of the bulk moduli $\kappa ^{\rm H}=(\mathbb {E}_{11}^{\rm H}+\mathbb {E}_{12}^{\rm H}+\mathbb {E}_{21}^{\rm H}+\mathbb {E}_{22}^{\rm H})/4$ and the Poisson’s ratio ratios $\mu ^{\rm H}=\mathbb {E}_{12}^{\rm H}/\mathbb {E}_{11}^{\rm H}$ of the 2D SGD are shown in panels a and b of Fig. 3. It can be seen that the Hashin–Shtrikman (HS) upper bound of the bulk modulus [50,51], expressed as ${v\kappa \mu }/ {[( {1 - v} ) + \mu ]}$, is achievable for the proposed SGD for all volume fractions; meanwhile, the Poisson’s ratio spans the range of $-0.8$ to 1. This provides sufficient candidates for mechanical metamaterials, making the proposed SGD a representative database, even though only unit cells with specific symmetry have been included so far. The representativeness and discussion of the 3D SGD are presented in Section D within the online supplementary material.

## APPLICATIONS

### Metamaterials with maximized bulk modulus

The proposed SGD is used to design mechanical metamaterials with maximum bulk modulus at $\bar{v} = 20\%, 30\%, 40\%$ and 50%, respectively. From different initial guesses, optimized designs obtained by the fast design model are shown in Fig. 3d. It can be seen that, under the same volume fraction constraint, the optimized designs have significantly different configurations yet close bulk modulus values. This demonstrates the richness of the proposed SGD and sets it apart from some databases containing only prototype unit cells of varying sizes [15,17]. Additionally, all relative errors between the predicted values and actual bulk moduli are smaller than 5%, validating the accuracy of the GCNN-based prediction model.

To further illustrate the representativeness of the constructed SGD, cross-shaped unit cells are compared to optimized unit cells obtained via inverse homogenization in Huang *et al.* [52]. As shown in Fig. 3c, both the optimized designs obtained by the SGD and the free-form designs obtained by topology optimization are very close to the 2D HS upper bounds of the bulk modulus. This validates the non-uniqueness of solutions and the representativeness of the proposed 2D SGD. The 3D unit cell with maximum bulk modulus is presented in Subsection G.1 within the online supplementary material. Additionally, 2D unit cells with maximized shear modulus are designed using the SGD, as detailed in Subsection H.1 within the online supplementary material.

Notably, since the optimized designs are described by explicit geometry parameters with a direct link to CAD/CAE software, it is easy to consider manufacturing uncertainties in the design process. In Subsection H.2 within the online supplementary material, a robust design formulation for metamaterials with maximum bulk modulus is proposed. The worst-case bulk modulus of the perturbed design can be significantly improved compared to its counterpart that does not consider manufacturing uncertainties.

### Auxetic metamaterials

**Figure 4.**
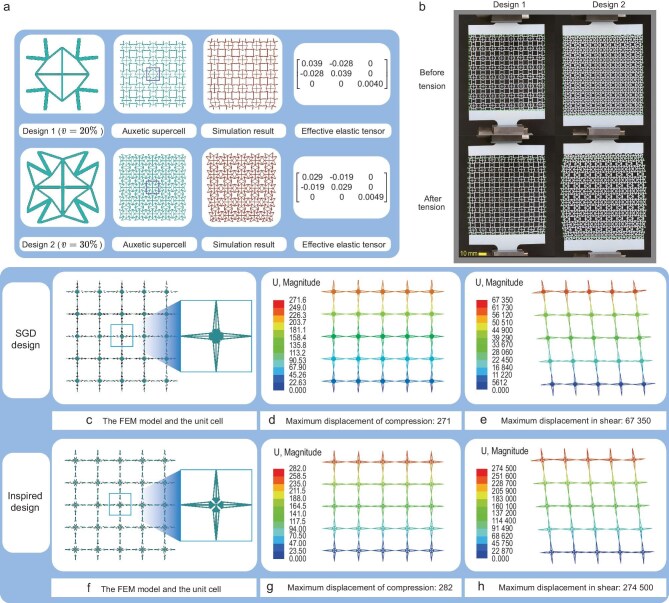
(a) The auxetic metamaterial designed by the 2D SGD. (b) The uniaxial tensile experiments verifying the auxetic behavior of SGD-based designs. (c–h) The unimode metamaterial generated by the 2D SGD and the inspired design generated by the SGD result.

By setting the objective function as $\mathbb {E}_{12}^{\rm H}/\mathbb {E}_{11}^{\rm H}$ and $\bar{v} = 20\%, 30\%$, solving the optimization problem (1) yields the optimized auxetic unit cells shown in Fig. 4a. It can be seen that, for all obtained designs, component $\mathbb {E}_{12}^{\rm H}$ is negative, implying that a stretch in the horizontal direction leads to an expansion in the vertical direction. Thanks to the explicit description in the MMC method, these designs were simulated in ABAQUS, and the auxetic behavior was verified, as shown in Fig. 4a (more details can be found in Subsection I.1 within the online supplementary material). Actually, the auxetic metamaterial designed by the SGD belongs to the so-called re-entrant auxetic structure [53], characterized by the concave inward bending of the unit cell’s edges. Under external forces, a leveraging effect occurs between unit-cell struts to the surrounding unit cells, exhibiting auxetic behavior. In addition, the cross structure at the center of obtained designs provides an effective stiffness enhancement. This is quite consistent with the specific treatment of adding extra ribs to produce stiff auxetic metamaterial [53].

Furthermore, $100\times 100\,{\rm mm}^2$ supercells composed of $8 \times 8$ unit cells were fabricated by additive manufacturing, as shown in Fig. 4b. To illustrate the auxetic behavior more clearly, green boxes identify the size of specimens before tension. It is evident that, under uniaxial tensile loading, both optimized designs exhibit pronounced auxetic behavior, as demonstrated in Fig. 4b. More details can be found in Section L within the online supplementary material. The 3D auxetic metamaterial is presented in Subsection G.2 within the online supplementary material.

### Unimode metamaterials

The so-called unimode material has a single easy mode of deformation, where one eigenvalue of the elasticity tensor is very small compared to the others [54]. In the constructed 2D SGD, due to the symmetry, we minimize component $\mathbb {E}_{33}^{\rm H}$ with the max allowable volume fraction $\bar{v} =$ 10% in optimization problem (1). As shown in Fig. 4c, the exterior of the optimized unit cell from the SGD design degenerates into hinges, consistent with the theoretical unimode material model composed of rigid bars and pivots proposed by Milton [55]. Using the geometry parameters, a $5 \times 5$ array of the optimized design is modeled in ABAQUS and analyzed under uniform distributed compressive and shear loads, with an applied concentrated force $F=1$ on the same top nodes of the supercell, and the bottom of the supercell fixed. More information about the finite-element analysis can be found in Subsection I.2 within the online supplementary material. The displacement contour plots corresponding to compressive and shear loads are shown in panels d and e of Fig. 4, respectively. As expected, due to the poor resistance of hinges for shear deformation, the maximum displacement amplitude under shear is about 248 times larger than the maximum displacement amplitude under compression. Furthermore, in Fig. 4f, we present an inspired design by introducing an additional hinge at the center of the SGD design result. The volume fractions of the SGD design and the inspired design are 9.3% and 9.4%, respectively. This modification further decreases the resistance to shearing deformation, and under the same external load, the shear deformation reaches as much as 3 orders of magnitude greater than the tensile/compressive deformation, indicating that the inspired design is closer to the theoretical unimode material. The pentamode metamaterial designed by the 3D SGD is presented in Subsection G.3 within the online supplementary material.

The above example suggests that, although the current SGD is somewhat restricted by the description of the $C_{4v}$-symmetric unit cells, it can nevertheless produce a rich family of mechanical metamaterials. Such a limitation can of course be alleviated by further introducing more general ‘structural genes’ into the SGD.

### Generalization of the SGD: buckling-resistant metamaterials

To investigate the generalizability of the SGD to non-local properties that can span across multiple unit cells, buckling behavior is considered as an example. Using the GCNN model and dataset described above, we employed transfer learning [56] to predict the critical buckling factor with only a relatively small amount of training data (approximately 7600 samples). For a detailed mechanical analysis of the unit cells and the training process of the GCNN model, the reader is referred to Subsection J.1 within the online supplementary material.

We further applied the buckling module of the 2D SGD to the optimization design of buckling-resistant metamaterials. Specifically, based on Bloch theory [57], the optimization formulation of a unit cell with maximum critical buckling load is expressed as


**Figure 5.**
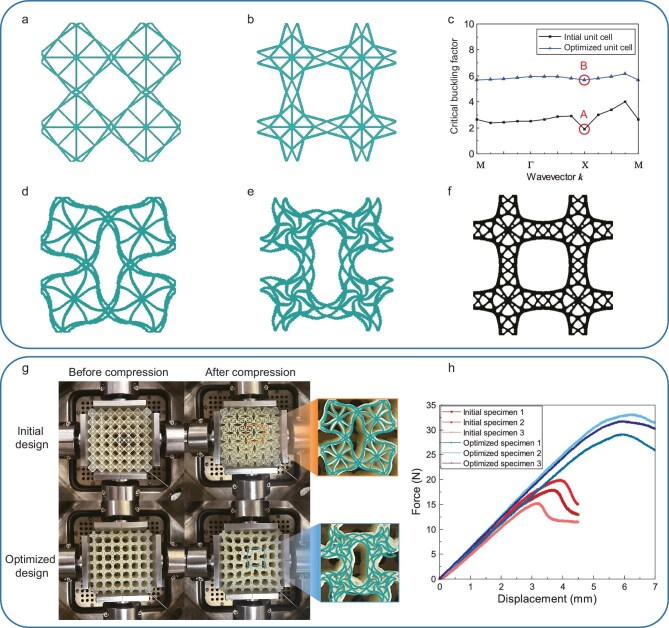
Design and experimental validation of a buckling-resistant metamaterial. (a) Initial unit cell (minimum critical buckling factor of 1.897). (b) Optimized design (minimum critical buckling factor of 5.668). (c) Critical buckling factors corresponding to different wavevectors of the initial and optimized designs. (d and e) Buckling modes corresponding to points A and B, respectively. (f) Reference design [58]. (g) Snapshots of supercells before and after the compression test (the enlarged views show that the simulated buckling modes coincide with the real deformation). (h) Force-displacement curves of the six additively manufactured specimens.

(3) \begin{eqnarray*}
\begin{array}{c}\text{find} \quad {\boldsymbol {d} \in \mathcal {D}_{ad}} \\
\text{min.} \quad {f = - {\min \limits _{\boldsymbol {k} \in \mathcal {B}}( {\bar{\alpha }( \boldsymbol {k} )} )}} \\
\text{s. t.} \quad {{\bar{\alpha }( \boldsymbol {k} )} = {\widetilde{\mathbb {G}}}( {\mathcal {G} \lbrace { {\boldsymbol V}({\boldsymbol d}), {\boldsymbol A}({\boldsymbol d})} \rbrace } )},\\
{V( \boldsymbol {d} ) \le \bar{v}}, \end{array}
\end{eqnarray*}

where $\bar{\alpha }( \boldsymbol {k} )$ denotes the critical buckling factor predicted by the generalized SGD with respect to the wavevector $\boldsymbol {k}$ and $\mathcal {B}$ denotes the boundary of the first irreducible Brillouin zone. Using PyTorch, the sensitivity information for the optimization problem above can be automatically obtained through the BP algorithm, which involves internal approximations for the min function. The initial unit cell shown in Fig. 5a has a minimum critical buckling factor of 1.897, while this value can be increased by 199% to 5.668 in the SGD-based optimization, as illustrated by Fig. 5b.

Compared to the initial design in Fig. 5a, the components distribute more uniformly in the optimized design in Fig. 5b and this yields a more uniform stress distribution with a smaller extreme amplitude. As a result, as depicted in Fig. 5c, the critical buckling factors for all wavevectors are significantly improved in the optimized design and the difference between the maximum and minimum values of $\bar{\alpha }$ decreases. Panels d and e of Fig. 5 respectively show the distinct buckling configurations of the initial design and the optimized design. This result is consistent with the topology optimization design results from [58], which reveal that, under biaxial compression, the unit cell with maximum critical buckling load is hierarchically structured (as shown in Fig. 5f), and that the critical buckling factors corresponding to different wavevectors tend to be consistent. Because of the limitations of available configurations in the present SGD, a complex design like Fig. 5f cannot be produced. However, the optimized design shown in Fig. 5b from the SGD and the topology optimization design shown in Fig. 5f are similar, indicating that the present optimized design successfully captured the ‘specialized feature’ of structural hierarchy.

To verify the effectiveness of the buckling-resistant design, we fabricated three groups of initial and optimized supercells using additive manufacturing. The specimens before and after uniaxial compression, along with the corresponding force-displacement curves, are presented in Fig. 5h. We find that the buckled configurations obtained experimentally are consistent with the numerical simulation results in Fig. 5g. The optimized buckling-resistant model exhibits a different buckling mode that enhances stability compared to the initial design. Specifically, the critical buckling loads of the initial model specimens are $17.93 \,{\rm N}$, $19.98 \,{\rm N}$, $15.18 \,{\rm N}$ with an average value of $17.7 \,{\rm N}$. In contrast, the critical buckling loads of the optimized specimens are $29.18 \,{\rm N}$, $33.14 \,{\rm N}$, $31.75 \,{\rm N}$, with an average value of $31.36 \,{\rm N}$, representing a 77.18% increase. These results validate the superior buckling resistance of the optimized design produced by the SGD. More details can be found in Section L within the online supplementary material.

### Generalization of the SGD: bandgap metamaterials

To verify the generalization ability of the proposed SGD approach for dynamic properties, we constructed a dispersion module using transfer learning and the well-trained GCNN model of the 2D SGD with approximately $11\, 000$ newly generated samples, to predict the normalized dispersion ($\bar{\omega } = \omega /\sqrt{{E\rho }/{(2(1 + \nu ))}}$ with $\omega$ and $\rho$ denoting the eigenfrequency and density). Details of the dataset generation and network training are provided in Subsection J.2 within the online supplementary material.

In the literature, maximizing the first-order bandgap is a common design objective for mechanical metamaterials [59]. Without loss of generality, the objective function is defined as


**Figure 6.**
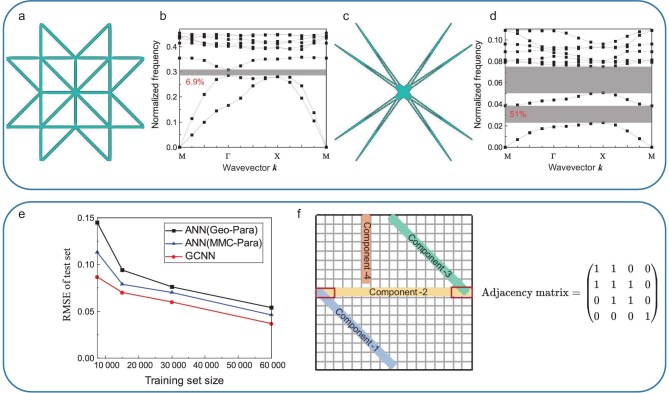
(a) A random initial unit cell and (b) the corresponding dispersion diagram. (c) The optimized unit cell and (d) the corresponding dispersion diagram. (e) Comparison of the prediction accuracy of different NN models with different dataset sizes. (f) An illustration of the adjacency matrix of 2D unit cells described by MMCs.

(4) \begin{eqnarray*}
f = 1 - \frac{{\bar{\omega }}_{2} - {\bar{\omega }}_{1}}{{\bar{\omega }}_{c}},
\end{eqnarray*}

where $[\bar{\omega }_{1},\bar{\omega }_{2}]$ denotes the range of the first-order bandgap identified from the predicted normalized dispersion, and $\bar{\omega }_{c} = (\bar{\omega }_{1}+\bar{\omega }_{2})/2$ is its center frequency. By minimizing the objective function (4) using GA as the optimizer and the well-trained dispersion module of the 2D SGD as a surrogate, from the random initial design illustrated in Fig. 6a, the optimized design illustrated in Fig. 6c was obtained.

In the optimized unit cell, a solid cross is located at the center, and eight groups of thin components connect the solid crosses between adjacent unit cells. This is a typical locally resonant metamaterial, which enables the bandgap in the low-frequency range. By reanalyzing the unit cell in Fig. 6c in ABAQUS, the normalized dispersion diagram of the initial design is plotted as Fig. 6d. Actually, the first-order bandgap is located at [0.0228,0.0384] with a relative bandgap width of $51.0\%$. In comparison, the dispersion diagram of the initial design is illustrated in Fig. 6b with a first-order bandgap located at [0.2860,0.3065] and a relative bandgap width of $6.9\%$. Besides, using deep learning–aided topology optimization [59], the relative bandgap width of an optimized phononic crystal with maximized first-order bandgap is $48.1\%$. Such a fact again demonstrates the representativeness of the 2D SGD in fast design of mechanical metamaterials.

## DISCUSSION

In this work, an SGD has been developed to establish the reciprocal mapping between geometry parameters and effective properties of a class of unit cells for the intelligent design of mechanical metamaterials. Two key strategies are utilized: the MMC-based explicit description of unit cells and the GCNN-based deep learning model. Using the MMC method, the proposed SGD contains unit cells with various topologies, making it richer than databases containing only specific prototype unit cells with varying sizes. Additionally, the explicit geometry description of unit cells facilitates metamaterial design in CAD/CAE without any postprocessing and reduces the number of design variables compared to methods with implicit topology descriptions, especially significant for the 3D SGD. The GCNN model leverages the geometrical connection between MMCs for feature extraction, leading to improved accuracy of the deep learning model.

To verify the effectiveness of the proposed strategies, two artificial neural networks with a similar architecture of fully connected layers as the GCNN model were trained on different dataset sizes simultaneously. The corresponding MSEs of the test sets are presented in Fig. 6e, indicating that the accuracy of all NN models increases with the number of training samples. Compared to the performance of the neural network model using the original geometry parameters, the MMC parameters provide more valuable information and improve prediction accuracy. The GCNN model yields the most accurate prediction of the effective elastic tensor for all datasets because it fully utilizes the adjacency matrix (i.e. the connection relation between MMCs) beyond the MMC parameters. It is expected that the prediction accuracy could be further improved with more training data. More details about the three NN models are presented in Section K within the online supplementary material.

Furthermore, even in the proposed structural genome database, the one-to-many mapping can make it difficult to train a regression neural network model to map a given elasticity tensor to a specific unit cell. Using the GCNN as a surrogate model, this inverse design problem can be solved efficiently. In principle, with the representativeness guaranteed, the proposed SGD can be used for any inverse design problems related to designing an architected material with a target elasticity tensor. The limitations of the established SGD mainly come from two aspects.

1. *Limited configurations of unit cells.* Because of predefined symmetry, the number of components, and the upper and lower bounds of design variables in producing optimal unit cells for various mechanical metamaterials, some configurations may always remain uncaptured by the SGD. Consequently, it cannot be guaranteed that the SGD can produce every kind of optimal mechanical metamaterial (e.g. optimal buckling-resistant metamaterials and 3D unit cells with maximum bulk modulus). However, our numerical examples demonstrate that even within a limited design space, optimization designs based on the structural genome database can still create highly ‘specialized features’ of the optimal unit cells (e.g. the expected hierarchical feature for a buckling-resistant metamaterial and plate-like features for maximizing the bulk modulus of a 3D unit cell). This shows that deep learning endows our structural genome database with ‘intelligence’ and could possibly guide the evolution of structural genes (introducing new structural genes with specialized features) towards optimal unit cells.
2. *Limitations on effective properties of metamaterials.* In this paper, we established a structural genome database based on the effective elasticity tensor, which cannot be directly applied to the design of mechanical metamaterials for other properties such as fracture resistance. To generalize the structural genome database to other properties, we may not need to completely retrain the GCNN model. By utilizing a small number of samples combined with transfer learning, the functionality of the structural genome database can be expanded. This is because the graph calculation part of the proposed neural network adequately explores the geometric information of the unit cells relatively independently of the prediction part. If the description of the unit cell remains unchanged within the structural genome database and only the predicted properties are modified, we can directly utilize the well-trained graph convolution calculation part and only update the fully connected layers. This approach also facilitates the future generalization of the structural genome database to include nonlinear properties of mechanical metamaterials.

Subsequent work will focus on improving the configuration of structural genes and developing multi-functional metamaterials based on the structural genome database in multiple physics fields.

## METHODS

### Unit cells described by the MMC method

In the MMC method [39,46], a set of components described by their geometry parameters (e.g. the center coordinate, length, width, inclined angles, etc.) is used to compose the optimized structures. For each component, a so-called topological description function (TDF; ${\phi }^i,\, i=1,\dots ,n$, with *n* denoting the number of components) is explicitly determined by its geometry parameters [46]. Furthermore, a unit cell in the SGD is similarly identified by the global TDF ${\phi }^{\rm s} = {\rm {max}}({\phi }^1,\dots ,{\phi }^{n})$ and the relations


(5) \begin{eqnarray*}
\left\{\begin{array}{ll}{\phi }^{\rm s}(\boldsymbol {x}) > 0 &\quad \text{if }\,\, \boldsymbol {x} \in {\Omega }_{s},\\
{\phi }^{\rm s}(\boldsymbol {x}) = 0 &\quad \text{if }\,\, \boldsymbol {x} \in \partial \rm {\Omega }_{s},\\
{\phi }^{\rm s}(\boldsymbol {x}) < 0 &\quad \text{if } \boldsymbol {x} \in {\rm {D}}\setminus ({\Omega }_{\rm s} \cup \partial \rm {\Omega }_{s}) , \end{array}\right.
\end{eqnarray*}

where $\rm {\Omega }_s$ and *D* are regions occupied by the solid material and the design domain of the unit cell, respectively.

To avoid re-meshing, a fixed mesh is adopted for the finite-element analysis. Under this circumstance, relations (5) are replaced by the regularized Heaviside function to determine the density of the solid material at each node of the finite elements:


(6) \begin{eqnarray*}
\rho &=& H_{\epsilon }^{\alpha }({\phi }^{\rm s} )\\
&=& \left\lbrace \begin{array}{@{}l@{\quad }l@{}}1& \text{if } x \ge \epsilon , \\
{\displaystyle\frac{3( {1 - \alpha } )}{4} \bigg ( {\displaystyle\frac{{\phi }^{\rm s}}{\epsilon } - \displaystyle\frac{({\phi }^{\rm s})^{3}}{3\epsilon ^{2}}} \bigg ) + \displaystyle\frac{1 + \alpha }{2}} & \text{if } |x| < \epsilon , \\
\alpha & \text{otherwise}. \end{array}\right. \\
\end{eqnarray*}

Here $\alpha = 10^{- 3}$ and $\epsilon = 0.2$. Young’s modulus of each finite element is determined by the ersatz material model as $E^{e} = ({E}/{M}){\sum _{i = 1}^{M}\rho _{i}^{e}}$ with $E,\,M$ and $\rho _{i}^{e}$ denoting Young’s modulus of the solid material, the number of nodes in each finite element and the *i*th nodal density of the *e*th element, respectively. Based on the density of each element, the volume fraction of each unit cell can be easily determined, and the effective elastic tensor is obtained through the asymptotic homogenization analysis in Section A within the online supplementary material.

### Graph structure of the unit cells described by MMCs

A graph structure $\mathcal {G}\lbrace \boldsymbol {V},\boldsymbol {E}\rbrace$ is composed of vertices and edges connecting vertices. The vertex matrix $\boldsymbol {V}=(\boldsymbol {V}^1,\boldsymbol {V}^2,\dots ,\boldsymbol {V}^n)$ contains information of the object, while the edge set $\boldsymbol {E}=\lbrace e(\boldsymbol {V}^1,\boldsymbol {V}^2 ),\ldots ,e(\boldsymbol {V}^{n-1},\boldsymbol {V}^n)\rbrace$ contains the specific relationship between any two objects. We denote by $e(\boldsymbol {V}^a,\boldsymbol {V}^b)$ the edge connecting two arbitrary vertices $\boldsymbol {V}^a,\boldsymbol {V}^b$. In particular, the adjacency matrix is usually used to store edge information and defined as


(7) \begin{eqnarray*}
A_{ij} = \left\lbrace \begin{array}{@{}l@{\quad }l@{}}1 & \text{if } e( {\boldsymbol {V}^{i},\boldsymbol {V}^{j}} ) \in \boldsymbol {E}, \\
0 & \text{otherwise}, \end{array}\right. \quad i,j \in [1,\dots ,n].
\end{eqnarray*}

For unit cells described by the MMC method, the vertices of the graph structure include the design variables of all the MMCs. In the 2D case, the *i*th vertex corresponds to $\boldsymbol {V}^{i}=(x_0^i,y_0^i,L^i,t^i,{\rm {sin}}\theta ^{i},{\rm {cos}}\theta ^{i})$. The adjacency matrix naturally denotes the connectivity between the MMCs and can be efficiently determined by


(8) \begin{eqnarray*}
A_{ij} = \left\lbrace \begin{array}{@{}l@{\quad }l@{}}1, &{\max ( {\min ( {{\boldsymbol {den}}_{i},{\boldsymbol {den}}_{j}} )} )} > 0, \\
0, & \text{otherwise}, \end{array}\right. \quad i \ne j,
\end{eqnarray*}

where ${\boldsymbol {den}}_{i},{\boldsymbol {den}}_{j}$ represent the density vectors of the *i*th and *j*th components on the fixed meshes, respectively, and ${\max ( {\min ( {{\boldsymbol {den}}_{i},{\boldsymbol {den}}_{j}} )} )} > 0$ indicates that the two MMCs are connected to each other. Figure 6f indicates the adjacency matrix of a representative unit cell.

## Supplementary Material

nwaf053_Supplemental_File
