# Supplementary material for: Intelligent design of mechanical metamaterials: a GCNN-based structural genome database approach
Source: Natl Sci Rev. 2025 Feb 20;12(4):nwaf053. doi: 10.1093/nsr/nwaf053 (PMC11912873; doi:10.1093/nsr/nwaf053)
Supplement: nwaf053_Supplemental_File [file nwaf053_supplemental_file.pdf]

Supplementary: Intelligent design of mechanical metamaterials: a GCNN-based structural genome database approach

Wenyu Hao[1†], Zongliang Du[1,2†], Xiuquan Hou[3], Yilin Guo[1], Chang Liu[1,2], Weisheng Zhang[1,2], Huajian Gao[4*], Xu Guo[1,2*]

[1*]State Key Laboratory of Structural Analysis, Optimization and CAE Software for Industrial Equipment, Department of Engineering Mechanics, Dalian University of Technology, Dalian, 116023, Liaoning, China.
[2] Ningbo Institute of Dalian University of Technology, Ningbo, 315016, Zhejiang, China.
[3]Institute of Artificial Intelligence and Robotics, Xi'an Jiaotong University, Xian, 710049, Shanxi, China.
[4*]Mechano-X Institute, Applied Mechanics Laboratory, Department of Engineering Mechanics, Tsinghua University, Beijing, 100084,China.

*Corresponding author(s). E-mail(s): gao.huajian@tsinghua.edu.cn; guoxu@dlut.edu.cn;
Contributing authors: haowenyu@mail.dlut.edu.cn; zldu@dlut.edu.cn; xiuqhou@stu.xjtu.edu.cn; guoyilin2018@mail.dlut.edu.cn; C.Liu@dlut.edu.cn; weishengzhang@dlut.edu.cn;
†These authors contributed equally to this work.

A Asymptotic homogenization analysis

According to asymptotic homogenization theory, the effective elastic tensor of a periodic composite can be calculated by the following equation [1]

$$\mathbb{E}^{\mathrm{H}}_{ijkl} = \frac{1}{|V|}\int_V \mathbb{E}_{pqrs}\left(\varepsilon^{0(ij)}_{pq} - \varepsilon^{(ij)}_{pq}\right)\left(\varepsilon^{0(kl)}_{rs} - \varepsilon^{(kl)}_{rs}\right)\mathrm{d}V \quad (1)$$

where $|V|$ is the volume of the unit cell, $\mathbb{E}$ and $\mathbb{E}^{\mathrm{H}}$ are the elastic tensors of the solid material and the homogenized phase, repsectively. The symbol $\varepsilon^{0(ij)}_{pq}$ is the unit

macroscopic strain fields, and $\boldsymbol{\varepsilon}_{pq}^{(ij)}$ is the linear stain of character displacement field $\boldsymbol{\chi}^{ij}$, i.e.,

$$\boldsymbol{\varepsilon}_{pq}^{(ij)} = \boldsymbol{\varepsilon}_{pq}\left(\boldsymbol{\chi}^{ij}\right) = \frac{1}{2}\left(\chi_{p,q}^{ij} + \chi_{q,p}^{ij}\right) \tag{2}$$

The characteristic displacement field $\boldsymbol{\chi}^{ij}$ can be obtained by solving the following equation:

$$\int_V \mathbb{E}_{ijpq}\varepsilon_{ij}(\boldsymbol{v})\varepsilon_{pq}\left(\boldsymbol{\chi}^{kl}\right)dV = \int_V \mathbb{E}_{ijpq}\varepsilon_{ij}(\boldsymbol{v})\varepsilon_{pq}^{0(kl)}\mathrm{d}V, \qquad \forall \boldsymbol{v} \in \mathcal{U}_{ad}^0 \tag{3}$$

where $\mathcal{U}_{ad}^0$ is the admissible set of virtual displacement field $\boldsymbol{v}$. In numerical implementation, the above equation systems are discretized by finite element method, and the open source Matlab code released by Andreassen et al. [2] was adopted. Specifically, the effective elastic tensor is calculated by the following discretized formula

$$\mathbb{E}_{ij}^{\mathrm{H}} = \frac{1}{|V|}\sum_{e=1}^{N}\left(\boldsymbol{\chi}_e^{0(i)} - \boldsymbol{\chi}_e^{(i)}\right)^{\top}\boldsymbol{k}_e\left(\boldsymbol{\chi}_e^{0(j)} - \boldsymbol{\chi}_e^{(j)}\right) \tag{4}$$

For the 2D case, the unit strains are defined as $\boldsymbol{\epsilon}^{(1)} = (1,0,0)^{\top}, \boldsymbol{\epsilon}^{(2)} = (0,1,0)^{\top}$ and $\boldsymbol{\epsilon}^{(3)} = (0,0,1)^{\top}$. In equation (4), $\boldsymbol{\chi}_e^{0(i)}$ contains the three nodal displacement fields of the e-th element corresponding to the unit strain $\boldsymbol{\epsilon}^{(i)}$, and $\boldsymbol{\chi}_e^{(j)}$ contains three columns corresponding to three displacement fields resulting from globally enforcing the unit strain $\boldsymbol{\epsilon}^{(j)}$. $\boldsymbol{k}_e$ is the element stiffness matrix.

B Generation of 2D unit cells for SGD based on the MMC method

In the structural genome database (SGD), all the 2D unit cells are assumed to be C_{4v}-symmetric. As shown in Fig. 1a, the 1×1 square design domain is divided into eight irreducible subdomains by the blue lines. Randomly select the control point $X_1(x_1, 0)$ on the edge AB, the control point $X_2(0.5, x_2)$ on edge OB, and the control point $X_3(x_3, x_3)$ on edge AO. Then connect the X_1X_2, X_2X_3, X_1X_3, X_2O, X_3O to form five initial components (green lines in Fig. 1a). The components in the other seven subdomains (yellow lines in Fig. 1a) can be determined according to the symmetry of the whole unit cell.

By randomly generating the coordinates of X_1, X_2, X_3 ($x_m \in (0, 0.5),\ m = 1,2,3$) and the half-widths of the five initial components as $t_n \in (0.001, 0.25),\ n = 1,2,...,5$, 2D unit cells can be produced according to Fig. 1a during the sampling process. Notably, these eight geometry parameters are the design variables of the unit cell, and they can be further converted to the moving morphable component (MMC) parameters [3, 4]. By locating a local Cartesian coordinate system $ox'y'$ at the center of the i-th component (see Fig 1b for reference), its topology description function (TDF) can be expressed as [4]:

$$\phi^i = 1 - \left[\left(\frac{x'}{L^i}\right)^p + \left(\frac{y'}{f(x')}\right)^p\right]^{\frac{1}{p}} \tag{5}$$

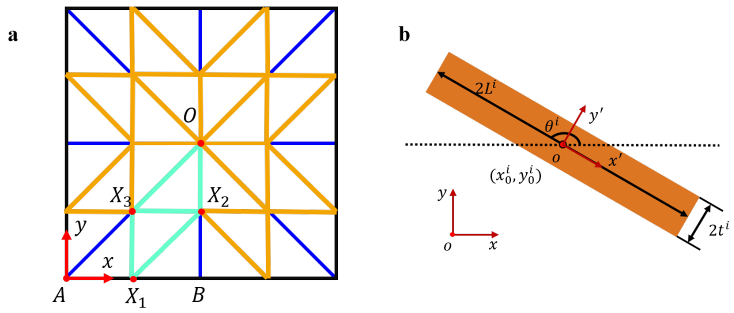

Fig. 1 **a** An illustration of the C_{4v}-symmetric unit cell described by the MMC method in the 2D SGD. **b** Geometry description of the i-th component in 2D case.

where $\begin{Bmatrix} x' \\ y' \end{Bmatrix} = \begin{bmatrix} \cos\theta^i & \sin\theta^i \\ -\sin\theta^i & \cos\theta^i \end{bmatrix} \begin{Bmatrix} y - y_0^i \\ x - x_0^i \end{Bmatrix}$ with θ^i denoting the inclined angle, p is a relatively large even number (e.g., $p = 6$), L^i is the half-length and $f(x') = t^i$ is the half-width of the i-th component, respectively. In particular, for the i-th component with coordinates of the endpoints as $P_j(x_j, y_j)$ and $P_k(x_k, y_k)$, its MMC parameters can be calculated as:

$$x_0^i = \frac{x_j + x_k}{2}, y_0^i = \frac{y_j + y_k}{2} \tag{6}$$

$$L^i = \sqrt{(x_j - x_k)^2 + (y_j - y_k)^2}/2 \tag{7}$$

$$\theta^i = \arctan\frac{y_k - y_j}{x_k - x_j} - \frac{\pi}{2}\left(\text{sign}\left(\arctan\frac{y_k - y_j}{x_k - x_j}\right) - 1\right) = \text{atan2}\,(y_k - y_j, x_k - x_j) \tag{8}$$

In the equation (8), sign is the symbolic function, and atan2 is a special arctangent function whose angle can be found in the whole quadrant, i.e.,

$$\text{sign}(x) = \begin{cases} 1 & \text{if } x > 0 \\ 0 & \text{if } x = 0 \\ -1 & \text{if } x < 0 \end{cases} \tag{9}$$

$$\text{atan2}\,(y, x) = \begin{cases} \arctan\frac{y}{x} & \text{if } x > 0 \\ \arctan\frac{y}{x} + \pi & \text{if } y \geq 0,\ x < 0 \\ \arctan\frac{y}{x} - \pi & \text{if } y < 0, x < 0 \\ +\frac{\pi}{2} & \text{if } y > 0, x = 0 \\ -\frac{\pi}{2} & \text{if } y < 0, x = 0 \\ \text{undefined} & \text{if } y = 0, x = 0 \end{cases} \tag{10}$$

By this means, the MMC parameters of 32 components in the 2D unit cells are obtained to calculate the density field following the method in the first subsection of Methods. The asymptotic homogenization analysis process in Section A is then used to produce the effective elastic tensor of the unit cells. Finally, the inclined angle θ in each component is converted to $\sin\theta$ and $\cos\theta$. Collecting $(x_0^i, y_0^i, L^i, t^i, \sin\theta^i, \cos\theta^i)^\top, i =$

1, ..., 32, results in a 32 × 6 feature matrix. This feature matrix, together with the adjacency matrix introduced in the second subsection of Methods, are set as the input to the graph convolutional neural network (GCNN).

C Generation of 3D unit cells for SGD based on the MMC method

In the generation process of the 1 × 1 × 1 3D unit cells, with cubic-symmetry assumed, nine different symmetric surfaces divide the design domain into 48 irreducible subdomains, as shown in Fig. 2. In the tetrahedral subdomain Ω_{ABCD}, by randomly setting the control points $N_1,\ N_2,\ N_3,\ N_4$ on the edge AB and face $\Gamma_{ABC},\ \Gamma_{BCD},\ \Gamma_{ACD}$ respectively, the connections $N_1N_2,\ N_1N_3,\ N_1N_4,\ N_2N_3,\ N_2N_4,\ N_3N_4$ are established as the MMCs in the design subdomain. These MMCs can be further used to generate 282 additional MMCs according to the symmetry.

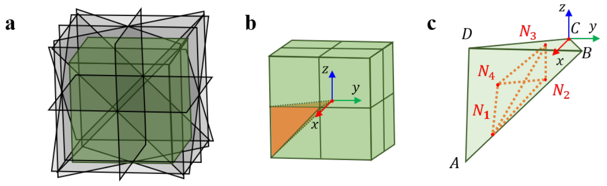

Fig. 2 **a** The cubic-symmetric 3D unit cells with its symmetric surfaces. **b** An irreducible subdomain as the design domain and **c** construction of MMCs in the design domain.

As illustrated by Fig. 2b and 2c, in particular, the locations of the four control points $N_1,\ N_2,\ N_3,\ N_4$ satisfy that:

$$\begin{aligned} &N_1: x_1 = 0.5, \quad y_1 = z_1 \in (-0.5, 0) \\ &N_2: \ x_2 \in (0, 0.5), \quad y_2 = z_2 \in (-0.5, 0) \\ &N_3: \ x_3 \in (0, 0.5), \ y_3 \in (-0.5, 0), \ z_3 = 0 \\ &N_4: \ x_4 = -y_4 \in (0, 0.5), \quad z_4 \in (-0.5, 0) \end{aligned} \tag{11}$$

In the present work, the 3D MMCs are restricted to be uniform rods with square cross section. By locating a local Cartesian coordinate system $ox'y'z'$ at the center of the i-th component, the corresponding TDF can be constructed as equation (12) [4]

$$\phi^i = 1 - \left[\left(\frac{x'}{L_1^i} \right)^p + \left(\frac{y'}{L_2^i} \right)^p + \left(\frac{z'}{L_3^i} \right)^p \right]^{1/p} \tag{12}$$

where $L_1^i,\ L_2^i,\ L_3^i$ denote the half-height, half-width and half-length of the i-th component, respectively. For all the 3D components, it satisfies $L_1^i = L_2^i \triangleq t_i \in (0.001, 0.25), i = 1, ..., 6$. With the help of Euler angles $\alpha^i,\ \beta^i,\ \gamma^i$, the coordinate

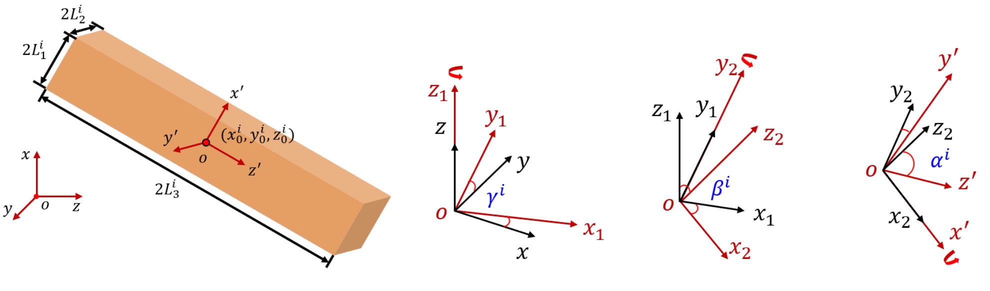

Fig. 3 **a** Geometry description of the i-th component in 3D case and **b** to **d** rotating the global coordinate system to the local coordinate system.

transformation between the local and global coordinate systems is defined as:

$$\begin{pmatrix} x' \\ y' \\ z' \end{pmatrix} = \begin{bmatrix} \cos\beta^i\cos\gamma^i & -\cos\alpha^i\sin\gamma^i + \sin\alpha^i\sin\beta^i\cos\gamma^i & \sin\alpha^i\sin\gamma^i + \cos\alpha^i\sin\beta^i\cos\gamma^i \\ \cos\beta^i\sin\gamma^i & \cos\alpha^i\cos\gamma + \sin\alpha\sin\beta^i\sin\gamma^i & -\sin\alpha^i\cos\gamma^i + \cos\alpha^i\sin\beta^i\sin\gamma^i \\ -\sin\beta^i & \sin\alpha^i\cos\beta^i & \cos\alpha^i\cos\beta^i \end{bmatrix}^\top \begin{pmatrix} x - x_0^i \\ y - y_0^i \\ z - z_0^i \end{pmatrix} \triangleq \boldsymbol{R}^i(\boldsymbol{x} - \boldsymbol{x}_0^i) \tag{13}$$

where x_0^i, y_0^i and z_0^i denote the center coordinates of the i-th component in the global Cartesian coordinate system. Similar as the 2D unit cells, the MMC parameters of all components can be solely determined by the geometry parameters. For the i-th component with vertexes $N_j\left(x_j, y_j, z_j\right)$ and $N_k\left(x_k, y_k, z_k\right)$, we have

$$x_0^i = \frac{x_j + x_k}{2},\ y_0^i = \frac{y_j + y_k}{2},\ z_0^i = \frac{z_j + z_k}{2} \tag{14}$$

$$L_3^i = \sqrt{\left(x_j - x_k\right)^2 + \left(y_j - y_k\right)^2 + \left(z_j - z_k\right)^2}/2 \tag{15}$$

On the other hand, the above rotation matrix can be expressed with the help of the so-called quaternions [5]. A quaternion is a hypercomplex number written as $\bar{q}_{bw} = q_0 + q_1\boldsymbol{i} + q_2\boldsymbol{j} + q_3\boldsymbol{k}$ with $q_0^2 + q_1^2 + q_2^2 + q_3^2 = 1$, where q_0, q_1, q_2, q_3 are real number, and $\boldsymbol{i}, \boldsymbol{j}, \boldsymbol{k}$ are mutually orthogonal vectors and satisfy the following definition:

$$\boldsymbol{i}^2 = \boldsymbol{j}^2 = \boldsymbol{k}^2 = -1, \boldsymbol{i}\times\boldsymbol{j} = \boldsymbol{k}, \boldsymbol{j}\times\boldsymbol{i} = -\boldsymbol{k}, \boldsymbol{j}\times\boldsymbol{k} = \boldsymbol{i}, \boldsymbol{k}\times\boldsymbol{j} = -\boldsymbol{i}, \boldsymbol{k}\times\boldsymbol{i} = \boldsymbol{j}, \boldsymbol{i}\times\boldsymbol{k} = -\boldsymbol{j} \tag{16}$$

The rotation matrix can be rewritten as [6]:

$$\boldsymbol{R} = \begin{bmatrix} q_0^2 + q_1^2 - q_2^2 - q_3^2 & 2\left(q_1q_2 - q_0q_3\right) & 2\left(q_0q_2 + q_1q_3\right) \\ 2\left(q_1q_2 + q_0q_3\right) & q_0^2 - q_1^2 + q_2^2 - q_3^2 & 2\left(q_2q_3 - q_0q_1\right) \\ 2\left(q_1q_3 - q_0q_2\right) & 2\left(q_0q_1 + q_2q_3\right) & q_0^2 - q_1^2 - q_2^2 + q_3^2 \end{bmatrix} \tag{17}$$

According to equation (13) to equation (17), the Euler angles of each component can be calculated as:

$$\gamma = \arctan\left(\tan\gamma\right) = \arctan\left(\frac{2\left(q_1q_2 + q_0q_3\right)}{1 - 2\left(q_2^2 + q_3^2\right)}\right) \tag{18}$$

$$\beta = \arcsin(\sin\beta) = \arcsin\left(2\left(q_0 q_2 - q_1 q_3\right)\right) \tag{19}$$

$$\alpha = \arctan\left(\tan\alpha\right) = \arctan\left(\frac{2\left(q_0 q_1 + q_2 q_3\right)}{1 - 2\left(q_1^2 + q_2^2\right)}\right) \tag{20}$$

Furthermore, q_0, q_1, q_2, q_3 of the quaternion can be determined by the vertex coordinates $N_j\left(x_j, y_j, z_j\right)$, $N_k\left(x_k, y_k, z_k\right)$ of a component. Let the direction of the vector $\overrightarrow{N_k N_j}$ be the z-axis direction of the component in the local coordinate system. The z-axis direction of the component before rotation is written as:

$$\boldsymbol{v}_1 = \left(0, 0, 1 + 2\mathrm{sign}\left(z_j - z_k\right)\right) \tag{21}$$

The z-axis direction of the rotated component is written as:

$$\boldsymbol{v}_2 = \left(x_j - x_k, y_j - y_k, z_j - z_k\right) \tag{22}$$

Then, the intermediate unit vector $\boldsymbol{h}$ of $\boldsymbol{v}_1$, $\boldsymbol{v}_2$ can be calculated by the follow equation:

$$\boldsymbol{h} = \left(\frac{\boldsymbol{v}_1}{\|\boldsymbol{v}_1\|} + \frac{\boldsymbol{v}_2}{\|\boldsymbol{v}_2\|}\right) / \left\|\frac{\boldsymbol{v}_1}{\|\boldsymbol{v}_1\|} + \frac{\boldsymbol{v}_2}{\|\boldsymbol{v}_2\|}\right\| \tag{23}$$

The real number q_0, q_1, q_2, q_3 of the quaternion can be calculated by the follow equations:

$$\begin{aligned} q_0 &= \boldsymbol{v}_1 \cdot \boldsymbol{h} \\ \left(q_1, q_2, q_3\right)^\top &= \boldsymbol{v}_1 \times \boldsymbol{h} \end{aligned} \tag{24}$$

With the 3D MMC parameters determined, cubic-symmetric 3D unit cells can be generated from the geometry parameters, as shown in Fig. 4. Similar to the 2D case, we used ersatz material model for the density field and calculated the effective elastic tensor by asymptotic homogenization analysis described in Section A.

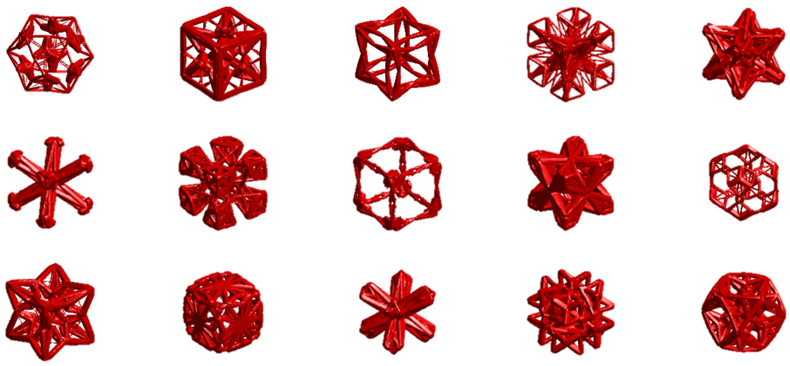

Fig. 4 Randomly generated 3D unit cell samples.

D Verification the representativeness of the 3D DSG

In the 3D case, the bulk modulus and shear modulus of the solid material is defined as $\kappa = \frac{E}{3(1-2\nu)}$ and $\mu = \frac{E}{2(1+\nu)}$, respectively. According to the Hashin-Shtrikman (H-S) bounding theoremIn the 3D case, the bulk modulus and shear modulus of the solid material is defined as $\kappa = \frac{E}{3(1-2\nu)}$ and $\mu = \frac{E}{2(1+\nu)}$, respectively. According to the Hashin-Shtrikman (H-S) bounding theorem [7, 8], the upper bound of the bulk modulus of the 3D unit cells in SGD is expressed as $\frac{4v\kappa\mu}{3(1-v)\kappa+4\mu}$. , the upper bound of the bulk modulus of the 3D unit cells in SGD is expressed as $\frac{4v\kappa\mu}{3(1-v)\kappa+4\mu}$.

With the effective elasticity tensors obtained, the bulk modulus can be calculated as

$$\kappa^{\mathrm{H}} = \frac{\mathbb{E}^{\mathrm{H}}_{11} + \mathbb{E}^{\mathrm{H}}_{12} + \mathbb{E}^{\mathrm{H}}_{13} + \mathbb{E}^{\mathrm{H}}_{21} + \mathbb{E}^{\mathrm{H}}_{22} + \mathbb{E}^{\mathrm{H}}_{23} + \mathbb{E}^{\mathrm{H}}_{31} + \mathbb{E}^{\mathrm{H}}_{32} + \mathbb{E}^{\mathrm{H}}_{33}}{9} \tag{25}$$

As shown in Fig. 5, the effective bulk modulus of 3D unit cells in the SGD does not reach the H-S upper bound. There are several reasons for this. Firstly, the cubic-symmetric unit cells generated following Fig. 2 greatly restrict the layout. Additionally, MMCs with square cross sections actually form truss-like unit cells, while it has been disclosed that plate-like unit cells are able to achieve the theoretical bounds of elastic properties [9]. In a forthcoming paper, more refined unit cells will be constructed for the 3D SGD.

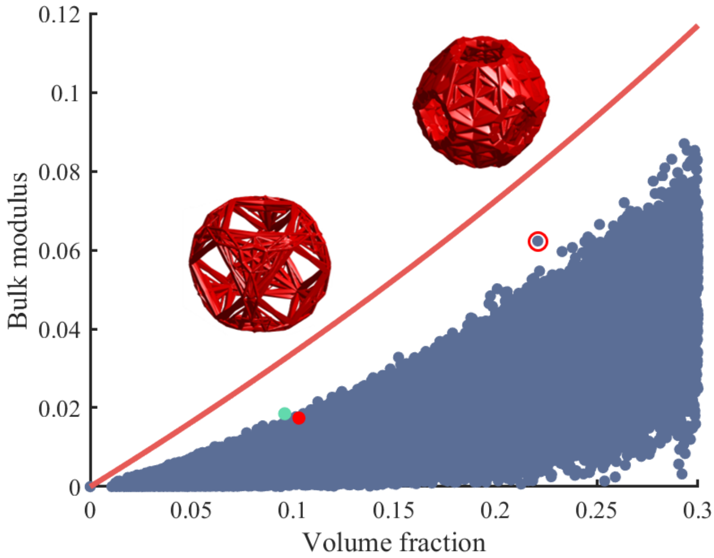

Fig. 5 Distribution of bulk modulus of the samples in the 3D SGD (Young's modulus $E = 1$ and Poission's ratio $\mu = 0.3$; green point - predicted performance, red point - actual performance).

E 2D and 3D GCNN model

E.1 Details of the GCNN model

The GCNN used in this study is composed of two main parts: the GCNII [10] message passing layers (MPL) and the fully connected layers. The $(l+1)$-th MPL is defined as:

$$\boldsymbol{H}^{(l+1)} = \sigma^{(l)} \left(\left((1-a_l)\, \tilde{\boldsymbol{D}}^{-\frac{1}{2}} \tilde{\boldsymbol{A}} \tilde{\boldsymbol{D}}^{-\frac{1}{2}} \boldsymbol{H}^{(l)} + a_l \boldsymbol{H}^{(0)} \right) \left((1-b_l)\, \mathbf{I} + b_l \boldsymbol{W}_d^{(l)} \right) \right) \tag{26}$$

where the hyperparameters are $a = 0.01$ and $b = \log(1/l)$, σ is the nonlinear activation function (Leaky ReLU is used for all hidden layers in the present work), $\tilde{\boldsymbol{D}} = \boldsymbol{D} + \mathbf{I}$ with $\boldsymbol{D}$ being the diagonal degree matrix, $\tilde{\boldsymbol{A}} = \boldsymbol{A} + \mathbf{I}$ with $\boldsymbol{A}$ being the adjacency matrix, $\mathbf{I}$ is the identity matrix and $\boldsymbol{W}_d$ is the weight matrix. The initial graph structure and weight matrix are expressed as the residual $\boldsymbol{H}^{(0)}$.

In the implementation process the MPL is rewrite as:

$$\boldsymbol{H}^{(l+1)} = \sigma^{(l)} \left((1-b_l)\, \boldsymbol{R}_1 + b_l \boldsymbol{R}_2 \boldsymbol{W}_d^{(l)} \right) \tag{27}$$

where $\boldsymbol{R}_1 = [\boldsymbol{H}^{(l)}; \boldsymbol{H}^{(0)}]$ and $\boldsymbol{R}_2 = (1-a_l)\, \tilde{\boldsymbol{D}}^{-\frac{1}{2}} \tilde{\boldsymbol{A}} \tilde{\boldsymbol{D}}^{-\frac{1}{2}} \boldsymbol{H}^{(l)} + a_l \boldsymbol{H}^{(0)}$. The fully connected layer is defined as:

$$\boldsymbol{Y}^{(k)} = \sigma^{(k)} \left(\boldsymbol{W}^{(k)} \boldsymbol{Y}^{(k-1)} + \boldsymbol{b}^{(k)} \right) \tag{28}$$

where $\boldsymbol{W}$ and $\boldsymbol{b}$ are the weight matrix and bias vector. The loss function adopted is the mean-square error (MSE)

$$Loss = \frac{\sum_{i=1}^{m} \left(Y_i^{\text{pred}} - Y_i^{\text{true}} \right)^2}{m} \tag{29}$$

where $\boldsymbol{Y}^{\text{pred}}$ and $\boldsymbol{Y}^{\text{true}}$ are the predicted output vector and the corresponding simulated result by homogenization procedure respectively. The symbol m is the dimension of the output vector, $m = 5$ for 2D and $m = 10$ for 3D respectively.

The deep learning training process was performed by using the Pytorch toolbox [11] on a PC with two Intel(R) Xeon(R) Gold 6246R CPU @ 3.40GHz CPU (with 512GB RAM) and an NVIDIA RTX A6000 GPU (with 48GB memory). The initial learning rate of the Adam optimizer was set to 5×10^{-2} and decayed by 20% every 80 epochs. The mini-batch size for the training was set to 512. The architectures of the GCNN models for the 2D SGD and the 3D SGD are listed in the Table 1 and Table 2, respectively.

E.2 Training details of the 3D SGD

Similar to the 2D case, collecting the $(x_0^i, y_0^i, z_0^i, L_1^i, t^i, \sin\alpha^i, \cos\alpha^i, \sin\beta^i, \cos\beta^i, \sin\gamma^i, \cos\gamma^i)^\top$, $i = 1, ..., 288$ forms a 288×11 matrix. This matrix, combined with the adjacency matrix of MMCs, form a graph structure as the input to the GCNN model.

Description	Input shape	Output shape
Message passing layer 1	32×6	32×6
Message passing layer 2	32×6	32×6
Message passing layer 3	32×6	32×6
Flatten	32×6	192
Fully connected layer 1 + Leaky Relu	192	2048
Fully connected layer 2 + Leaky Relu	2048	2048
Fully connected layer 3 + Leaky Relu	2048	2048
Fully connected layer 4	2048	5

Table 1 Architecture of the GCNN model for the 2D SGD.

Description	Input shape	Output shape
Message passing layer 1	288×11	288×11
Message passing layer 2	288×11	288×11
Message passing layer 3	288×11	288×11
Tensor concatenate	$3 \times (288 \times 11)$	$3 \times 288 \times 11$
Flatten	$3 \times 288 \times 11$	9504
Fully connected layer 1 + Leaky Relu	9504	2048
Fully connected layer 2 + Leaky Relu	2048	2048
Fully connected layer 3 + Leaky Relu	2048	2048
Fully connected layer 4 + Leaky Relu	2048	2048
Fully connected layer 5	2048	10

Table 2 Architecture of the GCNN model for the 3D SGD.

The volume fraction v and the non-zero components of the effective elastic tensor of the cubic-symmetric unit cells are recorded as the output vector, i.e., $\boldsymbol{Y} = (v, \mathbb{E}^{\mathrm{H}}_{11}, \mathbb{E}^{\mathrm{H}}_{12}, \mathbb{E}^{\mathrm{H}}_{13}, \mathbb{E}^{\mathrm{H}}_{22}, \mathbb{E}^{\mathrm{H}}_{23}, \mathbb{E}^{\mathrm{H}}_{33}, \mathbb{E}^{\mathrm{H}}_{44}, \mathbb{E}^{\mathrm{H}}_{55}, \mathbb{E}^{\mathrm{H}}_{66})^{\top}$.

As shown in Table 2, the Graph Convolutional Network via Initial residual and Identity mapping (GCNII) is combined with the fully-connected layers for prediction. By randomly sampling the coordinates of control points and the half-widths of the components, we obtained 100, 000 3D samples. These samples were divided into training, validation, and test sets by the ratio of 8:1:1. The comparisons between the actual and predicted outputs of the test set are shown in Fig. 6. Although the prediction accuracy is not as good as the 2D results in Fig. 2d-2h in the main text, the relationship between the geometry parameters and the effective properties of the 3D unit cells has been successfully extracted. Considering that the 3D GCNN model has a more complex output and input while the set of 3D samples is smaller (100,000 vs 240,000), it is expected that the prediction accuracy of the 3D GCNN model can be improved by increasing the number of training data.

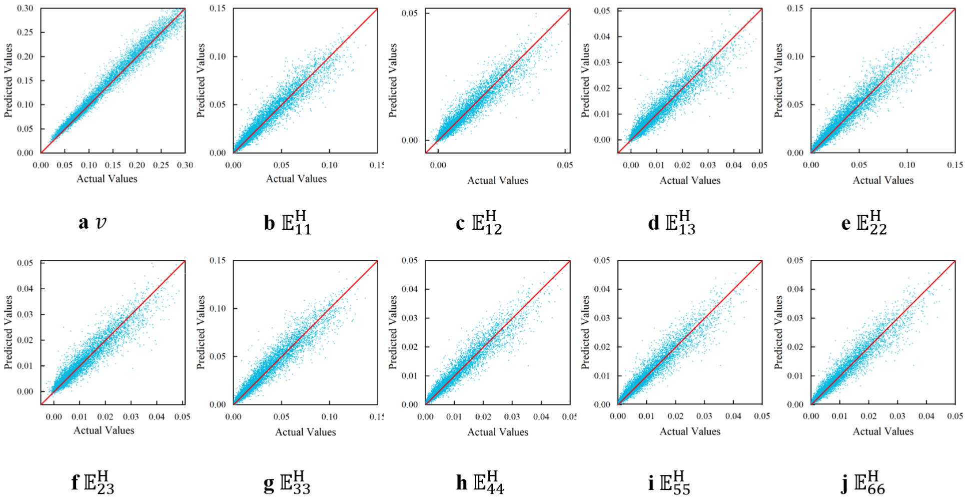

Fig. 6 The actual output vs the predicted output of the 3D GCNN model.

F Computational efficiency evaluation of the SGD

In this paper, we aimed to establish an efficient reciprocal relationship between geometry parameters and effective properties, with the core of acceleration lying in the GCNN model's prediction efficiency. Here, we compare the time cost of effective properties predicted by the SGD model and analyzed by asymptotic homogenization (AH) in Table 3. All the tests are completed on the same PC with two Intel(R) Xeon(R) Gold 6246R CPUs @ 3.40GHz (with 512GB RAM) and an NVIDIA RTX A6000 GPU (with 48GB memory). It can be seen that the prediction efficiency is accelerated by 1050 to 38,700 times for 2D and 3D unit cells.

Time cost	GCNN model (s)	AH analysis (s)	Acceleration factor
2D SGD	0.002	2.10	1050
Buckling module of 2D SGD	0.05	425.8	8516
Dispersion module of 2D SGD	0.08	306.2	3827
3D SGD	0.012	464.5	38700

Table 3 Comparison of time cost between SGD model and asymptotic homogenization analysis for various effective properties.

G Mechanical metamaterials design by the 3D SGD

To demonstrate the design capabilities of 3D SGD, we have conducted designs of unit cells with maximum bulk modulus, auxetic metamaterial, and pentamode metamaterial.

G.1 Metamaterials with maximized bulk modulus

Setting the maximum admissible volume fraction as $\bar{v} = 10\%$, a unit cell with maximum bulk modulus in the 3D SGD is shown in Fig. 7a. The predicted and actual properties are presented in Table 4 and scattered in Fig. 5. The green point corresponds to the predicted performance, while the red point corresponds to the actual performance of the optimized design.

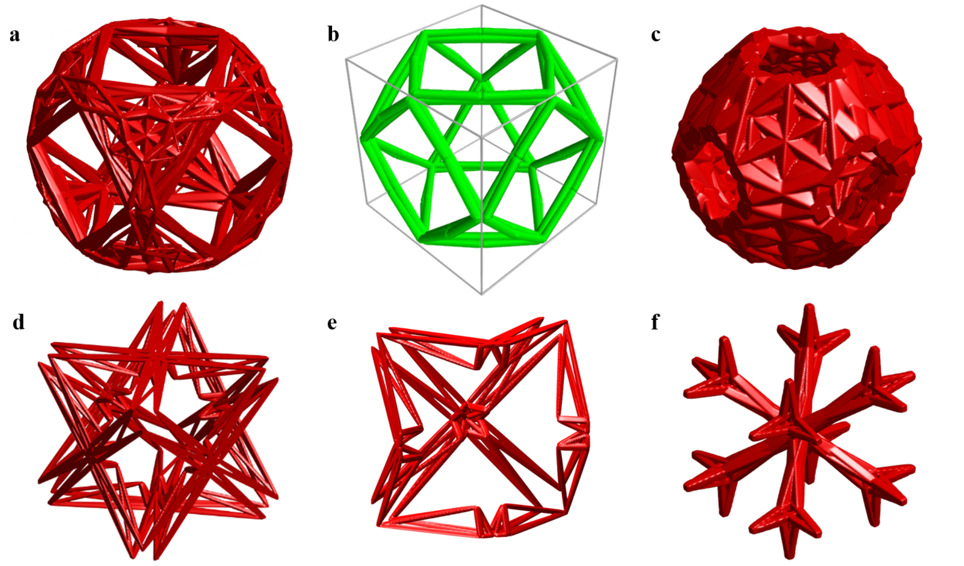

Fig. 7 **a** An illustration of the maximum bulk modulus design by the 3D SGD, **b** the maximum bulk modulus design of turss-like unit cell in reference [12], **c** the unit cell in the Fig. 5 circled in red, **d** the auxetic metamaterial design by 3D SGD, **e** the cross-sectional view of the auxetic unit cell and **f** the pentamode metamaterial design by 3D SGD.

We illustrate the maximum bulk modulus design of a truss-like unit cell (volume fraction is 5%) from Watt et al. [12], and the outlier sample point marked by the red circle in Fig. 5 in the Fig. 7b and 7c, respectively. The unit cells in the Fig. 7a and 7b have similar structures, both taking the form of the so-called octahedral rectified cubic truss. The unit cell in Fig. 7c forms a plate-like unit cell, resulting in a bulk modulus that is clearly higher than other truss-like unit cells. This demonstrates again that, even though the optimal bounds cannot be achieved due to the limitations on the configuration of structural genes, the SGD can still produce the specialized features of optimal unit cells.

G.2 Auxetic metamaterials

Setting the objective function as $\mathbb{E}_{12}/\mathbb{E}_{11}$ and a volume constraint of $v \leq 5\%$, using the 3D SGD, an optimized 3D auxetic metamaterial is shown in the Fig. 7d, with the

	$\mathbb{E}_{11}^{\mathrm{H}}$	$\mathbb{E}_{12}^{\mathrm{H}}$	$\mathbb{E}_{13}^{\mathrm{H}}$	$\mathbb{E}_{22}^{\mathrm{H}}$	$\mathbb{E}_{23}^{\mathrm{H}}$	$\mathbb{E}_{33}^{\mathrm{H}}$	$\mathbb{E}_{44}^{\mathrm{H}}$	$\mathbb{E}_{55}^{\mathrm{H}}$	$\mathbb{E}_{66}^{\mathrm{H}}$	v	Bulk modulus	Error
Predicted	0.027	0.014	0.014	0.027	0.014	0.027	0.012	0.012	0.012	0.096	0.0185	
Actual	0.026	0.013	0.013	0.026	0.013	0.026	0.010	0.010	0.010	0.103	0.0174	6.32%

Table 4 Predicted and actual properties of optimized unit cell with maximum bulk modulus using the 3D SGD.

corresponding Poisson's ratio as $\nu_{12} = -0.36$. As illustrated by Fig. 7e, all the faces of the 3D auxetic unit cell are the same, as a 2D re-entrant auxetic configuration, to induce the 3D auxetic behavior of the optimized unit cells. Such novel structural form differs from the common 3D auxetic metamaterials [13].

G.3 Pentamode metamaterials

Pentamode metamaterial is a novel 3D mechanical metamaterial featured by unusual elastic property of vanishing shear modulus like water. Similar as the inverse design of pentamode metamaterial based on AH analysis, the design objective is to maximize the ratio between the unit cell's bulk modulus and shear modulus, under a volume upper bound of $\bar{v} = 5\%$. In particular, for anisotropic unit cells, their bulk modulus is defined as equation (25) and shear modulus is defined as:

$$G^{\mathrm{H}} = \frac{\left(\mathbb{E}_{11}^{\mathrm{H}} + \mathbb{E}_{22}^{\mathrm{H}} + \mathbb{E}_{33}^{\mathrm{H}}\right) - \left(\mathbb{E}_{11}^{\mathrm{H}} + \mathbb{E}_{22}^{\mathrm{H}} + \mathbb{E}_{33}^{\mathrm{H}}\right) + 3\left(\mathbb{E}_{44}^{\mathrm{H}} + \mathbb{E}_{55}^{\mathrm{H}} + \mathbb{E}_{66}^{\mathrm{H}}\right)}{15} \tag{30}$$

The 3D SGD design is shown in the Fig. 7f and $\kappa^{\mathrm{H}}/G^{\mathrm{H}} = 10.69$. The "tentacle-like" connectors form hinges between unit cells, and this yields that the metamaterial has a weaker shear resistance compared to its compressive strength. Obviously, by refining those connectors, the ratio $\kappa^{\mathrm{H}}/G^{\mathrm{H}}$ can be further increased to enhance the pentamode property.

H Mechanical metamaterials design by 2D SGD

H.1 2D unit cells with maximized shear modulus

In the effective elastic tensor, the component $\mathbb{E}_{33}^{\mathrm{H}}$ represents the effective shear modulus. Using the fast design model in the SGD, unit cells with maximum shear modulus were obtained for maximum allowable volume fractions $\bar{v}$ as 25%, 35%, and 45%. Table 5 shows the predicted and actual shear moduli of these optimized designs in the 2D SGD, with all relative errors below 5%. Additionally, the designs were compared with the free-form design results from Huang et al. [14], as illustrated in Fig. 8. The performances of the present optimized designs are similar to the reference designs, although some errors in the predicted volume fractions caused deviations in the scatter plots in Fig. 8.

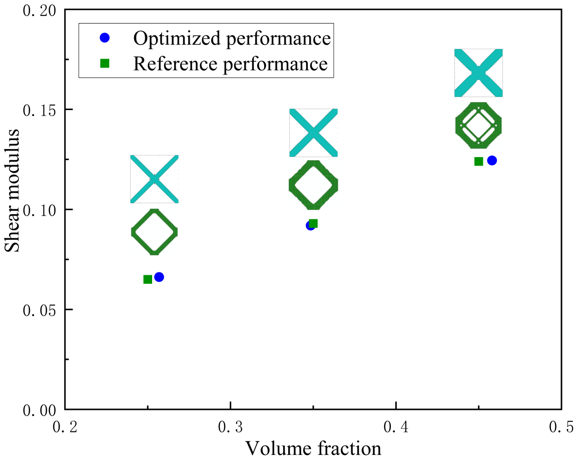

Fig. 8 Validation of the optimized unit cells with maximized shear modulus in the 2D SGD. The optimized performance corresponds to the exact shear modulus obtained by homogenization analysis and the exact volume fraction.

Prediction	0.0686	0.0960	0.1271
Truth	0.0662	0.0919	0.1244
Relative error	3.63%	4.46%	2.17%

Table 5 Error analysis of shear modulus of maximized shear modulus designs with different volume fractions.

H.2 Robust design of metamaterials with maximum bulk modulus considering manufacturing uncertainties

To reduce the sensitivity of the design results to manufacturing imperfections, we increased and decreased each variable by 5%, resulting in two new sets of design variables. The original design variables represent the ideal design, while the other two account for potential manufacturing errors or defects [15]. Taking the maximum bulk modulus design as an example, the design objective is modified to maximize the minimum bulk modulus among the three designs. This approach ensures that the bulk modulus is optimized under the most unfavorable conditions, thus ensuring the robustness of the obtained ideal design. Specifically, the corresponding optimization formulation is given by

$$\begin{array}{ll} \text{find} & \boldsymbol{d} \in \mathcal{D}_{\text{ad}} \\ \text{min.} & f = \max\left(-\bar{\kappa}_{\text{e}}(\boldsymbol{d}_{\text{e}}) \;\; -\bar{\kappa}(\boldsymbol{d}) \;\; -\bar{\kappa}_{\text{d}}(\boldsymbol{d}_{\text{d}})\right) \\ & V(\boldsymbol{d}) \leq \bar{v} \\ \text{s.t.} & \boldsymbol{d}_{\text{e}} = 0.95 \cdot \boldsymbol{d} \\ & \boldsymbol{d}_{\text{d}} = 1.05 \cdot \boldsymbol{d} \end{array} \tag{31}$$

where $\boldsymbol{d}$ represents the vector of design variables, $\mathcal{D}_{\mathrm{ad}}$ is the admissible set of the vectors of design variables and the perturbed design variables are denoted as $\boldsymbol{d}_e$ and $\boldsymbol{d}_{\mathrm{d}}$. The symbols $\bar{\kappa_{\mathrm{e}}}$, $\bar{\kappa}$ and $\bar{\kappa}_{\mathrm{d}}$ represent the bulk modulus of the unit cell corresponding to different design variables.

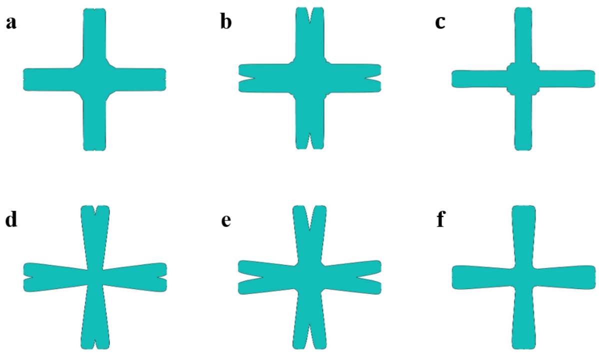

Fig. 9 Optimized unit cells with maximum bulk modulus: **a** the ideal design without considering robustness, **b** the perturbed design 1 without considering robustness (5% reduction in design variables), **c** the perturbed design 2 without considering robustness (5% increase in design variables), **d** the ideal design with considering robustness, **e** the perturbed design 1 with considering robustness (5% reduction in design variables) and **f** the perturbed design 2 with considering robustness (5% increase in design variables).

Fig. 9 presents the unit cells with the maximum bulk modulus with- and without robustness considerations, along with their perturbed designs. Table 6 provides the volume fractions and the corresponding bulk moduli of these configurations. Starting from the same initial design, the performance of the maximum bulk modulus design, considering robustness, is slightly lower (Fig. 9d, bulk modulus 0.0762), compared to the maximum bulk modulus design without considering robustness (Fig. 9a, bulk modulus 0.0891). However, the design performance considering robustness is more stable (see the Table 6 below). The most unfavorable bulk modulus only decreases by 2.0% (0.0747), whereas the most unfavorable bulk modulus without considering robustness is only 0.0645 (with a decrease of 27.6% compared to the ideal design).

	unit cells	Volume fractions	Bulk moduli	Changes
Without considering robustness	Ideal design	29.3%	0.0892	-
	Perturbed design 1	35.3%	0.1093	22.5%
	Perturbed design 2	22.8%	0.0646	−27.6%
Considering robustness	Ideal design	29.1%	0.0762	-
	Perturbed design 1	33.4%	0.0925	21.3%
	Perturbed design 2	26.7%	0.0747	−2.0%

Table 6 The impact of design variable perturbation on the bulk modulus.

I Finite element analysis of mechanical metamaterials

To verify the mechanical performance of the metamaterial designed by the SGD, finite element simulation is implemented in ABAQUS 6.14. A supercell composed of 5×5 unit cells is assembled and discretized using plane stress elements (CPS4R). The solid material is set with a Young's modulus $E = 1$ and Poission's ratio $\mu = 0.3$.

I.1 Simulation model of auxetic metamaterials

Fig. 10 illustrates the auxetic behavior of a supercell, where the displacement in the x-direction and y-direction at the bottom are fixed and uniform tensile loads with amplitude of 1 are applied on the top of the supercell.

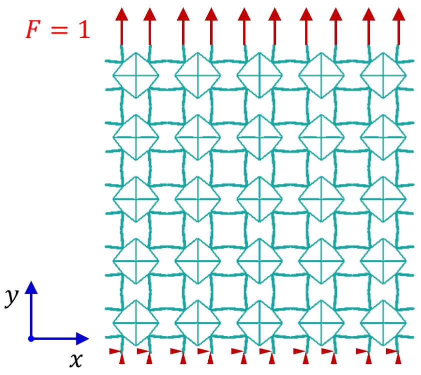

Fig. 10 Boundary conditions for the supercell of auxetic metamaterial.

I.2 Simulation model of unimode metamaterials

To verify the anisotropic load resistance of the unimode material, the supercells composed of 5×5 SGD designs and inspired designs are fixed at the bottom and loaded under uniformly distributed compressive and shearing loads with an amplitude of 1, as Fig. 11, respectively.

J Generalization of 2D SGD for non-local mechanical properties

J.1 Buckling module of the 2D SGD

Since buckling configurations could span multiple unit cells, the periodic boundary conditions of the unit cell are modified to Bloch boundary condition below

$$\boldsymbol{u}(x) = \boldsymbol{u}\left(x + \boldsymbol{a}\right) e^{-i\boldsymbol{k}\boldsymbol{a}} \tag{32}$$

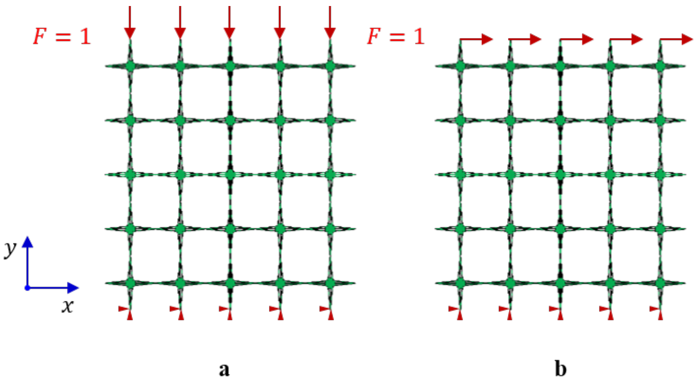

Fig. 11 Boundary conditions for the supercell of unimode metamaterial under **a** uniform compressive loads and **b** uniform shearing loads.

where $\boldsymbol{u}$ is the nodal displacement, $\boldsymbol{a} = (a_1, a_2)^\top$ is the lattice vector (corresponding to the length of the unit cell) and $\boldsymbol{k} = (k_1, k_2)^\top$ is the wavevector. As ABAQUS cannot directly handle complex-valued qurestions, it becomes necessary to separate all field variable $\boldsymbol{w}$ into their real part and imaginary part $\boldsymbol{w} = \boldsymbol{w}^{\mathrm{Re}} + i \cdot \boldsymbol{w}^{\mathrm{Im}}$ [16]. Therefore, the Bloch boundary condition in equation (32) is rewritten as follows

$$\begin{bmatrix} \boldsymbol{u}^{\mathrm{Re}}(x) \\ \boldsymbol{u}^{\mathrm{Im}}(x) \end{bmatrix} = \begin{bmatrix} \cos(\boldsymbol{ka}) & \sin(\boldsymbol{ka}) \\ -\sin(\boldsymbol{ka}) & \cos(\boldsymbol{ka}) \end{bmatrix} \begin{bmatrix} \boldsymbol{u}^{\mathrm{Re}}(x + \boldsymbol{a}) \\ \boldsymbol{u}^{\mathrm{Im}}(x + \boldsymbol{a}) \end{bmatrix} \tag{33}$$

Equation (33) can be applied across two unit cells using a user-defined subroutine in ABAQUS. Subsequently, stability analysis is performed to evaluate the buckling behavior of the structure.

Following the literature [17, 18], to investigate the critcal buckling behavior, we discretize the boundary of the first Brillouin zone into 12 different wavevectors, and calculate their critical buckling factors $\bar{\alpha}_1, \cdots, \bar{\alpha}_{12}$ under the loading conditions shown in Fig. 12b.

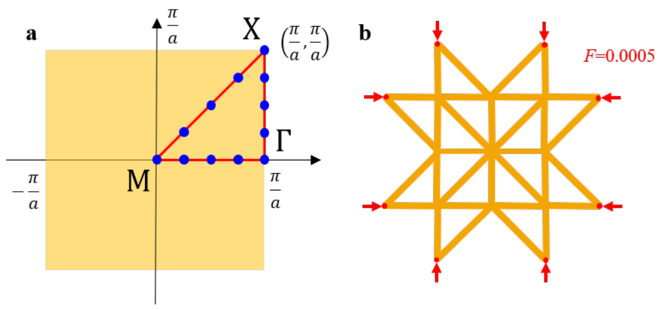

Fig. 12 **a** The first irreducible Brillouin zone and the sampling points that the wavevector $\boldsymbol{k}$ is taken for a C_{4v} symmetry unit cell and **b** the loading conditions for stability analysis of the unit cell.

According to Section B, 8477 unit cells were randomly generated, and the critical buckling factors $\bar{\alpha}_1, \cdots, \bar{\alpha}_{12}$ for each unit cell were calculated by ABAQUS 6.14. Similar to the existing SGD, the feature matrix containing MMC parameters, combined with the adjacency matrix forming the graph structure, serves as input parameters for the GCNN. The output consists of the volume fraction of the unit cell v and the critical buckling factor corresponding to different wavevectors $\boldsymbol{k}$, denoted as $\boldsymbol{Y} = \{v, \bar{\alpha}_1, \cdots, \bar{\alpha}_{12}\}^\top$.

During the training process, the sample set was divided into training, validation, and test sets in a ratio of 8:1:1. Due to the limited number of samples and the increased complexity of stability analysis compared to static analysis, we employed transfer learning for the GCNN model [19]. In fact, the network architecture shown in Fig. 2c in the main text can be divided into two parts. The graph convolution calculation part thoroughly explores the geometric information of the unit cell, while the later fully connected network part is used to predict the properties of interest. Therefore, if the description of the unit cell in the SGD remains unchanged and only the predicted properties are altered, we can freeze the parameters of the graph convolution calculation part (which have been well-trained using 240,000 data points). Subsequently, by training the network model with new samples, we can ensure better predictive performance with fewer samples. Table 7 shows the architecture of the GCNN model for the buckling module of 2D SGD. We freeze the parameters of MPL1 to MPL3, and the comparison of the differences in training the GCNN model directly and using transfer learning is shown in Fig. 13.

Description	Input shape	Output shape
Message passing layer 1	32×6	32×6
Message passing layer 2	32×6	32×6
Message passing layer 3	32×6	32×6
Message passing layer 4	32×6	32×6
Message passing layer 5	32×6	32×6
Message passing layer 6	32×6	32×6
Flatten	32×6	192
Fully connected layer 1 + Leaky Relu	192	2048
Fully connected layer 2 + Leaky Relu	2048	1028
Fully connected layer 3 + Leaky Relu	1028	512
Fully connected layer 4	512	13

Table 7 Architecture of the GCNN model for the buckling module of 2D SGD.

J.2 Bandgap module of the 2D SGD

Similar to the generalization of the SGD for long-wave instability property in Subsection J.1, the dispersion curves of bandgap metamaterials also rely on the Bloch boundary conditions. Discretizing the boundary of the first irreducible Brillouin zone

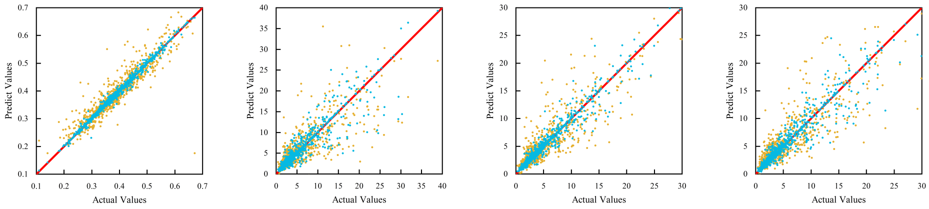

a b c d

Fig. 13 Comparison of direct training and transfer learning in predictive performance (yellow points represent results from direct training, blue points represent results using transfer learning techniques): **a** The volume fraction v, **b** the critical buckling factor $\bar{\alpha}_1$, **c** the critical buckling factor $\bar{\alpha}_{11}$ and **d** the critical buckling factor $\bar{\alpha}_{12}$.

by 12 discrete wavevectors, ten orders of eigenfrequencies are calculated to plot the dispersion diagram. According to Section B of the Supplementary Materials, 11,342 unit cells were randomly generated, and the frequency ω_j^i (where i denotes the order of the frequency $i = 1, \ldots, 10$ and j denotes the j-th wavevector, $j = 1, \ldots, 12$) for each unit cell were calculated by ABAQUS 6.14. Similar to the train process of the buckling module of the 2D SGD, we also employed transfer learning for the GCNN model (More details are listing in the Subsection J.1). Table 8 illustrates the corresponding architecture of the GCNN model for dispersion and the comparison of the actual and predicted values of the GCNN model is shown in Fig. 14.

Description	Input shape	Output shape
Message passing layer 1	32 × 6	32 × 6
Message passing layer 2	32 × 6	32 × 6
Message passing layer 3	32 × 6	32 × 6
Message passing layer 4	32 × 6	32 × 6
Message passing layer 5	32 × 6	32 × 6
Message passing layer 6	32 × 6	32 × 6
Flatten	32 × 6	192
Fully connected layer 1 + Leaky Relu	192	2048
Fully connected layer 2 + Leaky Relu	2048	1028
Fully connected layer 3 + Leaky Relu	1028	512
Fully connected layer 4	512	121

Table 8 Architecture of the GCNN model for the dispersion module of 2D SGD.

The genetic algorithm is implemented by using the optimization toolbox in Matlab 2020b. Related parameters are set as: the population size of 100, the crossover fraction of 0.2, the elite count of 5, the maximum generations of 500, the MaxStallGenerations of 5 and the fitness function tolerance of 10^{-3}.

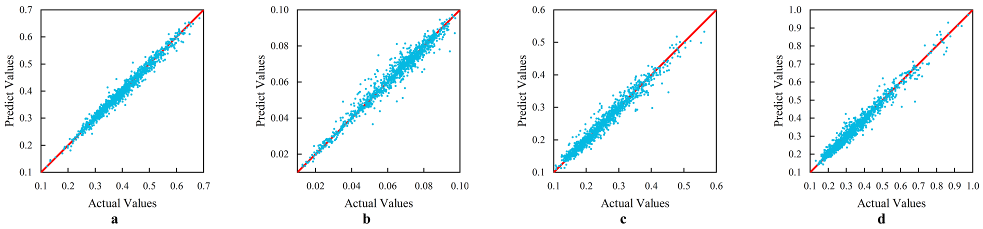

Fig. 14 The actual output vs the predicted output of the GCNN model for dispersion: **a** The volume fraction v; **b**, **c** and **d** are the frequencies ω_2^2, ω_6^6 and ω_{12}^{10} respectively.

K Validation of accuracy of the GCNN model

To verify the effectiveness of the proposed model, two artificial neural networks (ANN) with a similar architecture of fully connected layers as the GCNN model were trained simultaneously. In particular, as illustrated in Table 9, the ANN model denoted as ANN-Geo uses the geometry parameters as input, while the other model, ANN-MMC, uses the MMC parameters as its input.

	GCNN	ANN-Geo	ANN-MMC
Fully connected layer 1 + Leaky Relu	192	192	8
Fully connected layer 2 + Leaky Relu	512	512	512
Fully connected layer 3 + Leaky Relu	1024	1024	1024
Fully connected layer 4 + Leaky Relu	512	512	512
Fully connected layer 5	5	5	5

Table 9 Architectures of three different NN models.

By generating 60,000 samples, the performance of the three neural network (NN) models mentioned above is tested for different data sizes. Randomly taking 10% of the data as the test set, the NN models are trained three times, with their MSE values presented in Table 10. It can be observed that the GCNN model always has the smallest MSE values. Compared to the ANN-Geo and ANN-MMC models, the MSE of the GCNN model is improved by more than 25% and 10%, respectively. Furthermore, we used the multi-body dynamical sampling method proposed by Wu et al. [20] to generate a test set containing 6000 samples. The 2-norm errors between the predicted and actual values for each model are presented in Table 11. It is observed that the GCNN outperforms other two neural network models in terms of predictive accuracy.

Data Size	NN Model	MSE1	MSE2	MSE3	Differences
7500	ANN-Geo	0.1531	0.1454	**0.1451**	+66.21%
7500	ANN-MMC	0.1136	**0.1129**	0.1192	+29.32%
7500	GCNN	0.1021	0.0986	**0.0873**	-
15,000	ANN-Geo	**0.0942**	0.0966	0.0976	+33.43%
15,000	ANN-MMC	**0.0791**	0.0804	0.0804	+12.04%
15,000	GCNN	0.0713	0.0712	**0.0706**	-
30,000	ANN-Geo	0.0781	0.0799	**0.0758**	+27.82%
30,000	ANN-MMC	**0.0699**	0.0705	0.0705	+17.88%
30,000	GCNN	**0.0593**	0.0607	0.0607	-
60,000	ANN-Geo	**0.0544**	0.0546	0.0565	+45.07%
60,000	ANN-MMC	0.0466	**0.0455**	0.0467	+21.33%
60,000	GCNN	**0.0375**	0.0387	0.0394	-

Table 10 Mean-square errors of the three NN models trained by different data sets.

Error	v	$\mathbb{E}^H_{11}$	$\mathbb{E}^H_{12}$	$\mathbb{E}^H_{22}$	$\mathbb{E}^H_{33}$
ANN-Geo	1.19%	4.23%	5.53%	4.23%	4.75%
ANN-MMC	1.03%	3.55%	4.73%	3.55%	3.97%
GCNN	0.95%	3.06%	4.13%	3.06%	3.50%

Table 11 Comparison of the 2-norm prediction errors of different NN models for 6000 samples generated by the sampling method [20].

L Experimental verification

L.1 Fabrication

We fabricated each specimen using a Stratasys J35 Pro multi-material 3D printer with the digital material FLAXA95070, which has a shore hardness of 70. This digital material is a blend of three base materials: Elastico Clear, RGD531-DIGITALABS-PLUS, and RGD515-DIGITALABS-PLUS.

The supercells of auxetic metamaterials are constructed by 8×8 unit cells with dimensions of 100mm $\times$ 100mm $\times$ 3mm as Fig. 4b in the main text. The supercells of buckling-resistant metamaterials are constructed by 7×7 unit cells with dimensions of 100mm $\times$ 100mm $\times$ 42mm, as shown in Fig. 5g in the main text. The actual dimensions and weights of the six buckling-resistant metamaterial specimens are listed in Table 12.

	Specimen 1 - initial	Specimen 2 - initial	Specimen 3 - initial	Specimen 1 - optimized	Specimen 2 - optimized	Specimen 3 - optimized
Vertical length (mm)	101.8	101.6	101.4	100.4	102.1	101.8
Horizontal length (mm)	100.6	101.3	100.9	101.1	101.8	101.9
Thickness (mm)	42.1	42.0	42.2	42.0	42.1	42.2
Weight (g)	154.5	156.6	159.6	155.3	156.0	160.0

Table 12 Actual dimensions and weights of additive manufactured specimens.

L.2 Testing

During the tension test of auxetic metamaterial, all specimens were uniformly tensed by a TUOFENG TWF-200S at a speed of 0.15mm/min to alleviate the viscoelastic behavior of the fabricated specimens. In the test of buckling-resistant metamaterial, all specimens were uniformly biaxially compressed by a CARE Neo-10000 biaxial tester at a speed of 0.15mm/min. Note that the specific compression speed was determined by testing similar structures at different loading rates until the force-displacement curve achieved a rate-independent solution.

References

[1] Cheng, G., Cai, Y., Xu, L.: Novel implementation of homogenization method to predict effective properties of periodic materials. Acta Mech Sin **29**(4), 550–6 (2013)

[2] Andreassen, E., Andreasen, C.S.: How to determine composite material properties using numerical homogenization. Comput. Mater. Sci. **83**, 488–95 (2014)

[3] Guo, X., Zhang, W., Zhong, W.: Doing topology optimization explicitly and geometrically—a new moving morphable components based framework. J Appl Mech-Trans ASME **81**(8), 081009 (2014)

[4] Du, Z., Cui, T., Liu, C., Zhang, W., Guo, Y., Guo, X.: An efficient and easy-to-extend matlab code of the moving morphable component (MMC) method for three-dimensional topology optimization. Struct Multidiscip Optim **65**, 158 (2022)

[5] John, V.: Quaternion Algebras. Springer, Berlin/Heidelberg, Germany (2021)

[6] Henderson, D.M.: Euler angles, quaternions, and transformation matrices for space shuttle analysis. Technical report, NASA JSC Report (1977)

[7] Hashin, Z., Shtrikman, S.: A variational approach to the theory of the elastic behaviour of multiphase materials. J Mech Phys Solids **11**(2), 127–40 (1963)

[8] Torquato, S., Gibiansky, L., Silva, M., Gibson, L.: Effective mechanical and transport properties of cellular solids. Int J Mech Sci **40**(1), 71–82 (1998)

[9] Berger, J., Wadley, H., McMeeking, R.: Mechanical metamaterials at the

theoretical limit of isotropic elastic stiffness. Nature **543**(7646), 533–7 (2017)

[10] Chen, M., Wei, Z., Huang, Z., Ding, B., Li, Y.: Simple and deep graph convolutional networks. In: International Conference on Machine Learning, pp. 1725–35 (2020)

[11] Paszke, A., Gross, S., Massa, F., Lerer, A., Bradbury, J., Chanan, G., Killeen, T., Lin, Z., Gimelshein, N., Antiga, L., *et al.*: Pytorch: An imperative style, high-performance deep learning library. In: Advances in Neural Information Processing Systems, vol. 32, pp. 8026–37 (2019)

[12] Watts, S., Tortorelli, D.A.: A geometric projection method for designing three-dimensional open lattices with inverse homogenization. Int J Numer Methods Eng **113**(8), 1411 (2018)

[13] Li, X., Peng, W., Wu, W., Xiong, J., Lu, Y.: Auxetic mechanical metamaterials: from soft to stiff. Int J Extreme Manuf **5**(4), 042003 (2023)

[14] Huang, X., Radman, A., Xie, Y.M.: Topological design of microstructures of cellular materials for maximum bulk or shear modulus. Comput Mater Sci **50**(6), 1861–70 (2011)

[15] Silva, G.A., Beck, A.T., Sigmund, O.: Topology optimization of compliant mechanisms with stress constraints and manufacturing error robustness. Comput Meth Appl Mech Eng **354**, 397–421 (2019)

[16] Åberg, M., Gudmundson, P.: The usage of standard finite element codes for computation of dispersion relations in materials with periodic microstructure. J Acoust Soc Am **102**(4), 2007–13 (1997)

[17] Thomsen, C.R., Wang, F., Sigmund, O.: Buckling strength topology optimization of 2d periodic materials based on linearized bifurcation analysis. Comput Meth Appl Mech Eng **339**, 115–36 (2018)

[18] Brillouin, L.N.: Wave Propagation in Periodic Structure. Dover Publications, Mineola, N.Y. (1953)

[19] Yosinski, J., Clune, J., Bengio, Y., Lipson, H.: How transferable are features in deep neural networks? In: Advances in Neural Information Processing Systems, vol. 27, pp. 3320–28 (2014)

[20] Wu, F., Zhao, Y., Zhao, K., Zhong, W.: A multi-body dynamical evolution model for generating the point set with best uniformity. Swarm Evol Comput **73**, 101121 (2022)
